# Supplementary material for: LD Motif Recognition by Talin: Structure of the Talin-DLC1 Complex
Source: Structure. 2016 Jul 6;24(7):1130–41. doi: 10.1016/j.str.2016.04.016 (PMC4938799; doi:10.1016/j.str.2016.04.016)
Supplement: Document S1. Supplemental Experimental Procedures and Figures S1–S6 [file mmc1.pdf]

Structure, Volume *24*

Supplemental Information

LD Motif Recognition by Talin: Structure of the Talin-DLC1 Complex

Thomas Zacharchenko, Xiaolan Qian, Benjamin T. Goult, Devina Jethwa, Teresa B. Almeida, Christoph Ballestrem, David R. Critchley, Douglas R. Lowy, and Igor L. Barsukov

Supplementary information

LD-motif recognition by talin: structure of the talin-DLC1 complex

Thomas Zacharchenko, Xiaolan Qian, Benjamin T. Goult, Devina Jethwa, Teresa B. Almeida, Christoph Ballestrem, David R. Critchley, Douglas R. Lowy and Igor L. Barsukov

Supplementary Figures

Supplementary Methods

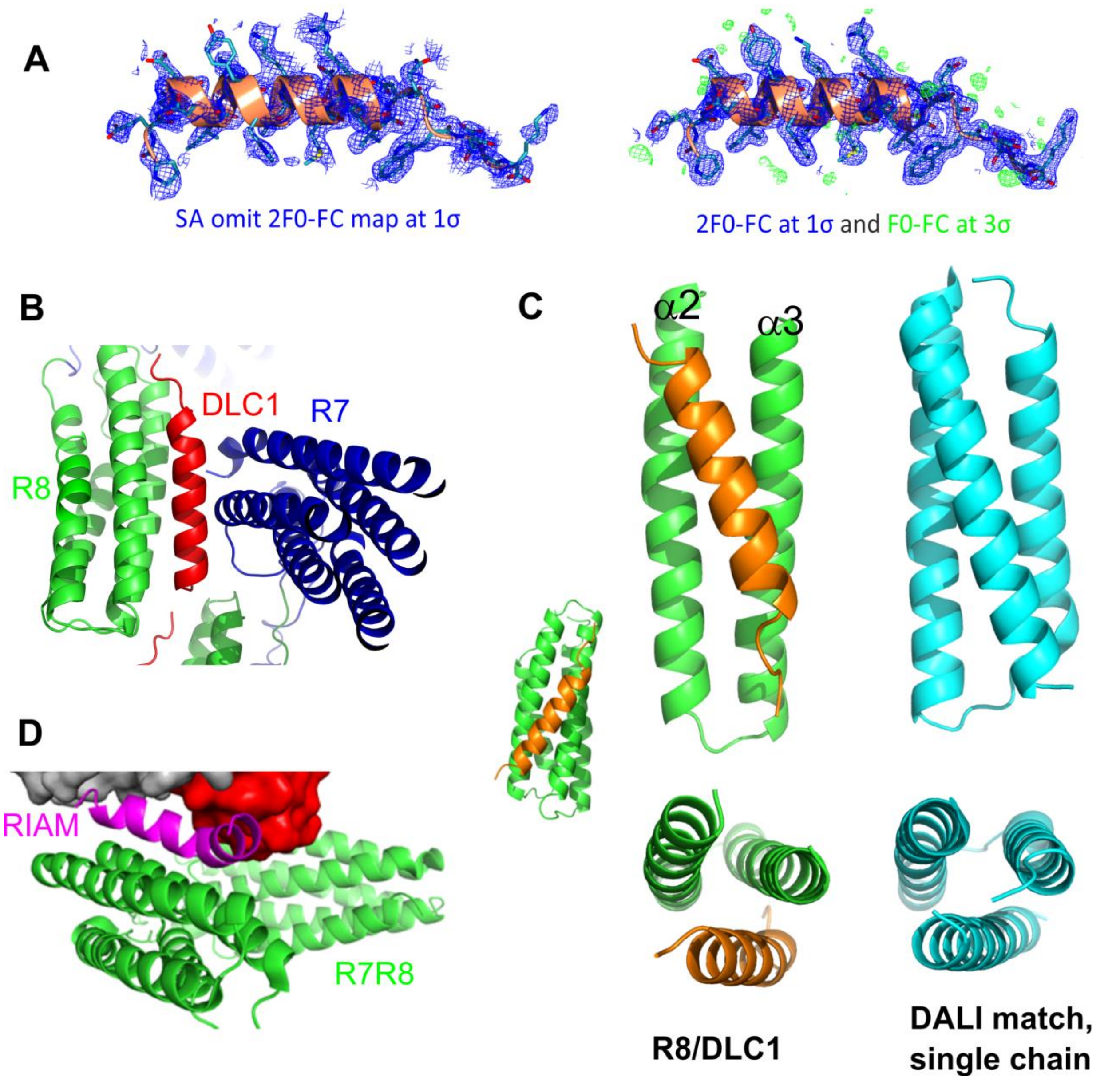

Figure S1, related to Figures 2 and 3. Structural characteristics of R7R8/DLC1 complex

(A) Structure of DLC1 peptide is well defined in the complex. Simulated annealing composite omit map 2F0-FC shown at 1 σ (blue, left) and the refined 2F0-FC map (blue, right) of the DLC1 LD-motif shown at 1 σ superimposed on the structure of the DLC1 peptide in the complex (orange) show a good match between the structure and the electron density.

(B) Crystal packing contacts in the R7R8/DLC1 complex. Outer surface of the DCL1 helix (red) makes limited contacts with the edge of the R7 domain of the symmetry-related molecule.

(C) Triple-helix structure in the talin R8/DLC1 complex. Similarity between the arrangement of the α2-α3 hairpin of the talin R8 and DLC1 fragment from the R8/DLC1 complex (left) and the single-chain, triple-helix structure of BAG family molecular chaperone regulator 4 (right; PDB: 4HWH), identified as a close match by DALI server (http://ekhidna.biocenter.helsinki.fi/dali_server/)

(D) Crystal packing contacts in the talin R7R8/RIAM complex (PDB: 4W8P). RIAM helix (magenta) bound to the R7R8 talin fragments (green) makes contacts with the symmetry-related talin R7 domain (red) that may induce the kink in the RIAM helix. A straight helix would clash with the symmetry-related R7.

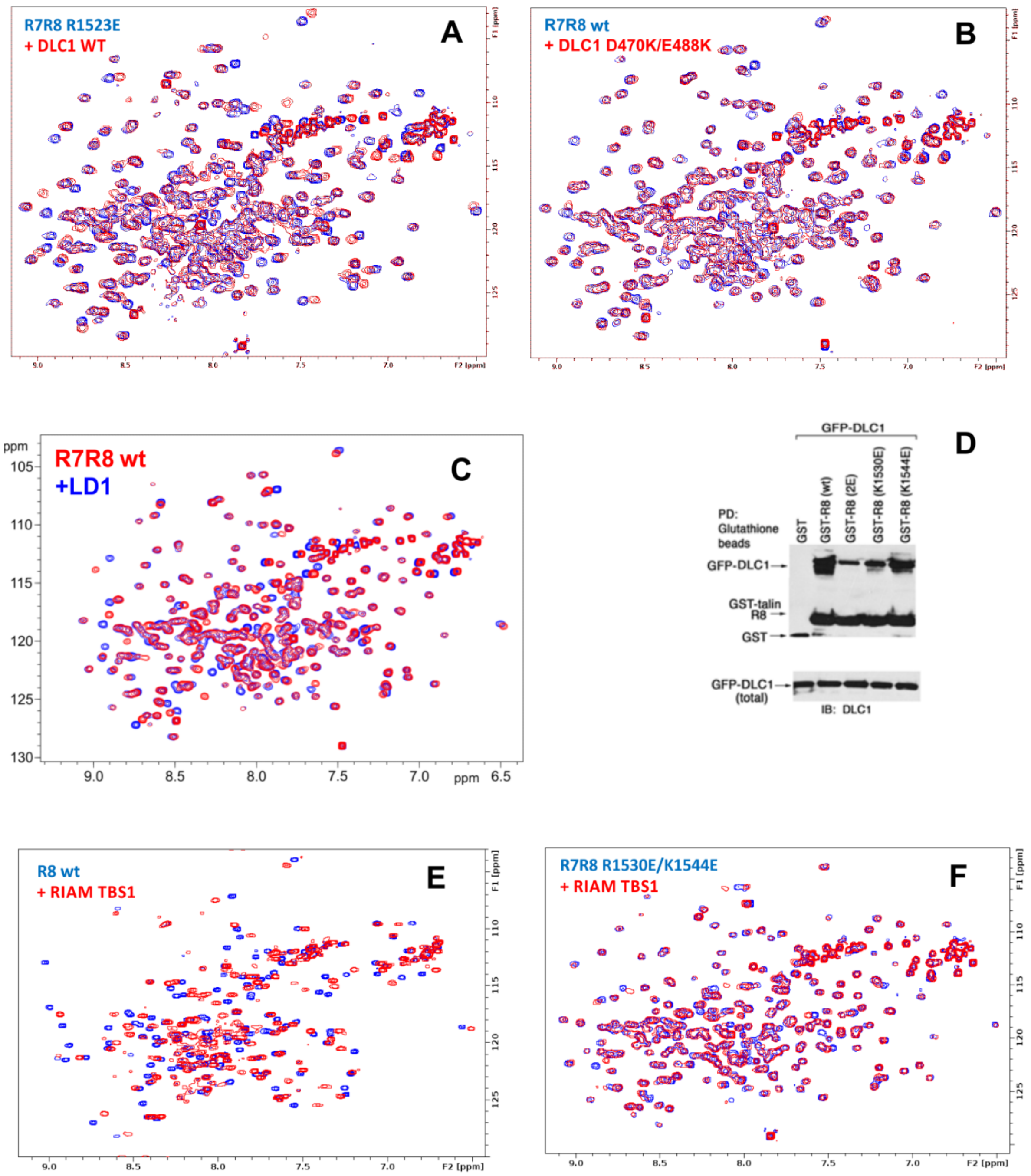

Figure S2, related to Figures 4 and 5. Point mutations disrupt the interaction between talin R8 and DLC1 or paxillin.

(A) Superposition of the HSQC spectra of 0.2 mM K1523E mutant of talin R7R8 free (blue) and in the presence of 0.8 mM DLC1(461-489) (red).

(B) Superposition of the HSQC spectra of 0.2 mM talin R7R8 free (blue) and in the presence of 0.8 mM D470K/E488K DLC1(461-489) mutant (red).

(C) Superposition of the HSQC spectra of 0.2 mM K1544E mutant of talin R7R8 free (red) and in the presence of 0.8 mM of paxillin LD1 (blue).

(D) GST-talin R1530E binds DLC1 less well than the K1544E mutant. Wild-type or mutant GST-R8 talin fragment were co-transfected with GFP-DLC1 into 293T cells. Cell extracts were subjected

to pull-down assay with glutathione beads followed by immunoblotting with anti-GST and anti-DLC1 on the same membrane (top). The transfected GFP-DLC1 in each sample is shown by the anti-DLC1 blot (bottom) as a loading control.

(E) Superposition of the HSQC spectra of 0.2 mM of talin R7R8 free (blue), and in the presence of 0.2 mM of RIAM TBS1 (red).

(F) Superposition of the HSQC spectra of 0.2 mM of R1530E/K1544E mutant of talin R7R8 free (blue), and in the presence of 0.2 mM of RIAM TBS1 (red).

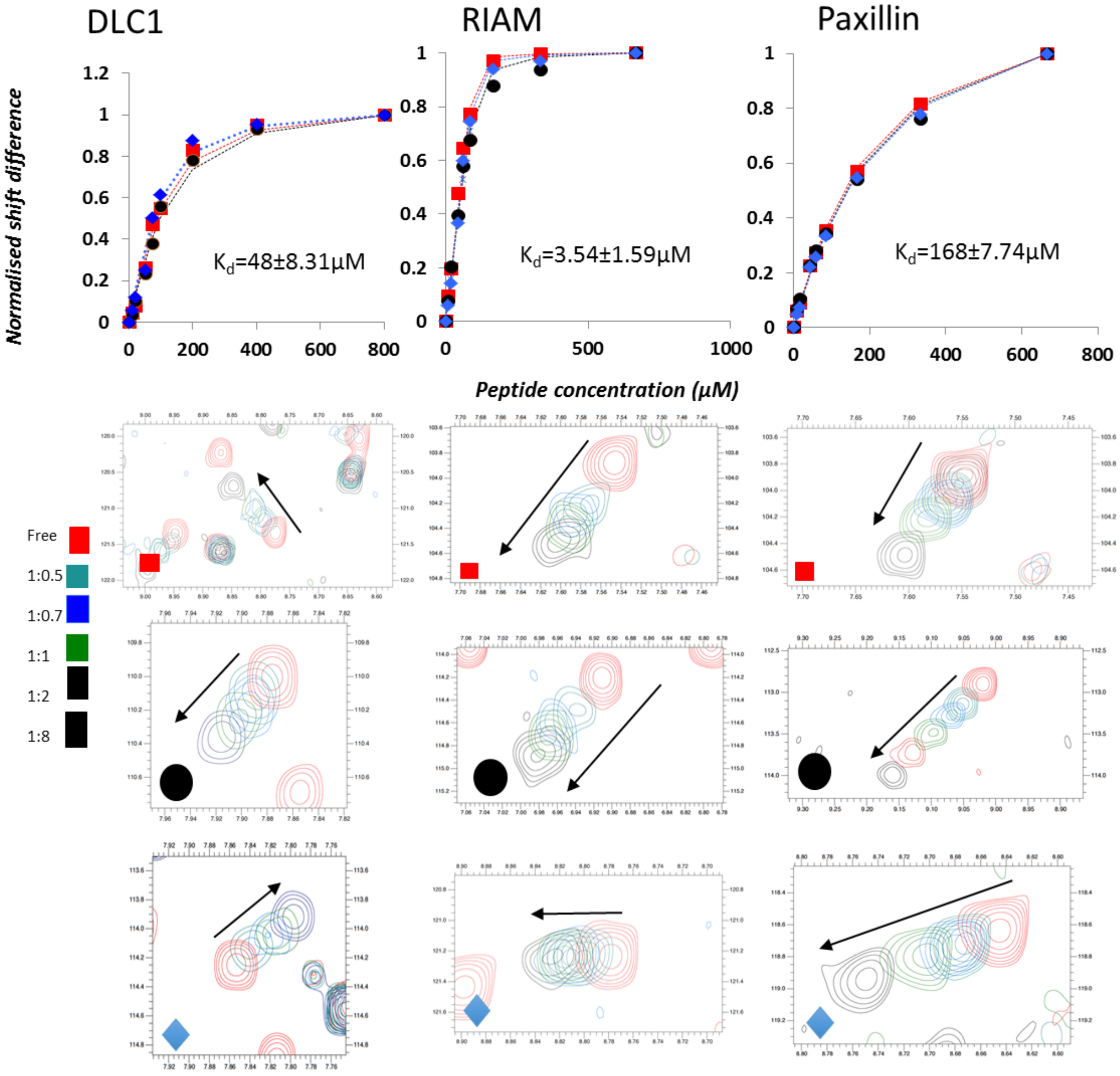

Figure S3, related to Figures 1 and 5. Dissociation constants for DLC1, RIAM and paxillin interactions with talin R8.

(A) Examples of the chemical shifts changes in the $^{1}H,^{15}N$-HSQC spectra of talin R8 on the addition of the peptide ligands used to determine K_d values. Symbols represent experimental chemical shift differences. Solid lines of the corresponding colours show the fitted curves. For each curve, chemical shift changes are normalised to the last value.

(B) Superposition of the HSQC spectra for each of the cross-peaks used in the fitting in (A). Limited resonance broadening throughout the titration confirms fast exchange regime for the cross-peaks used in the K_d measurements.

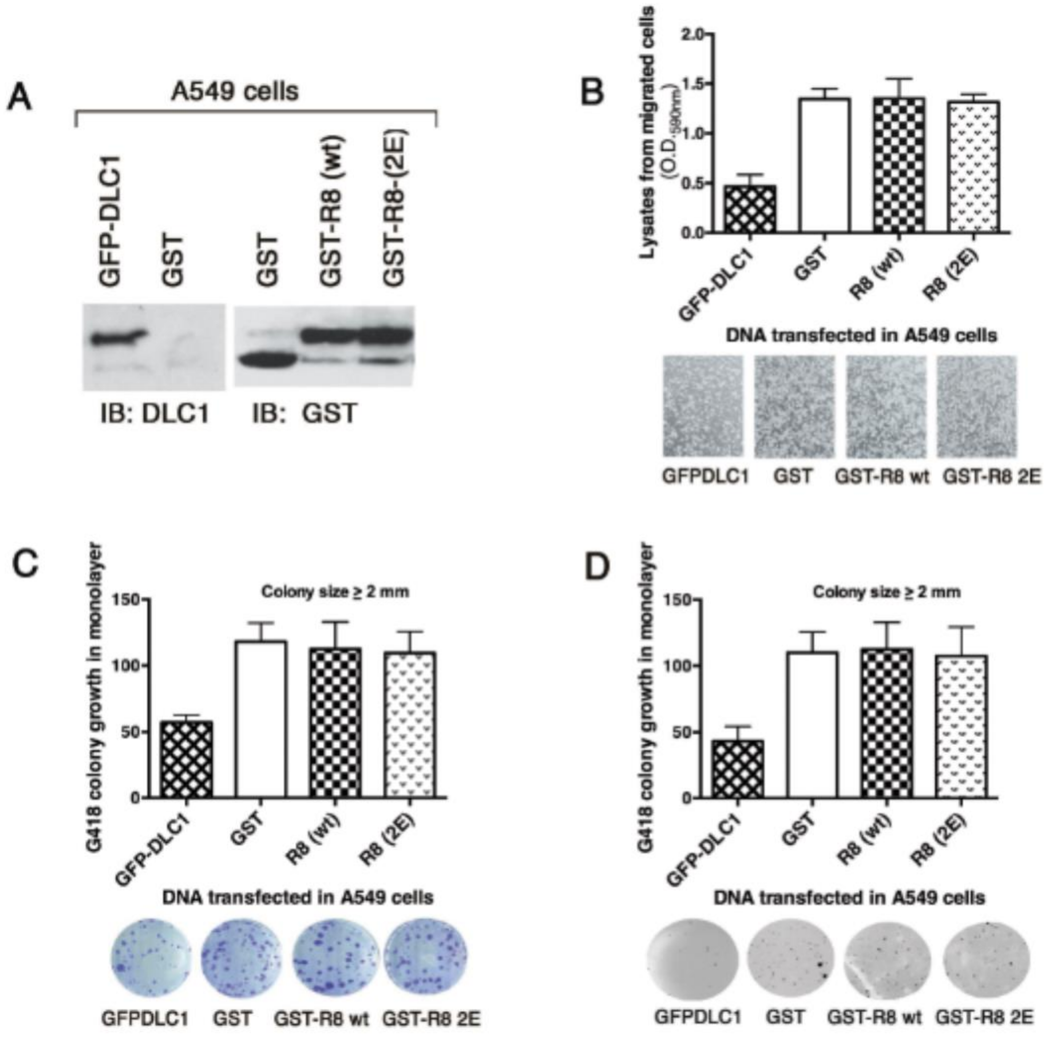

Figure S4, related to Figure 6. Expression of GST-talin R8 alone is insufficient to cause significant biological changes in A549 cells.

(A) Co-transfection of A549 cells with GFP-DLC1 and either GST, GST-R8 wt, or GST-R8 2E constructs. Six days after transfection, A549 cell extracts were analysed by anti-DLC1 (left) and anti-GST (right) immunoblotting (IB). Note – GST constructs were purified on glutathione beads prior to immunoblotting, and were expressed at similar levels.

(B) Cell migration. Transfected cells were analysed using a transwell migration assay as described in methods. The lysates from migrated cells were quantitated (top), and representative microscopic images of the migrated cells are shown (bottom). As expected, GFP-DLC1 suppressed cell migration whereas the the biological activity of the talin R8 wt and 2E mutant was similar to that of the GST negative control.

(C) G418 colony growth. The transfected cells were cultured in G418 for 3 weeks, and the colonies were counted and quantitated (top). Representative stained colonies are shown (bottom). As expected, GFP-DLC1 suppressed colony formation whereas the biological activity of the talin R8 wt and 2E mutant was similar to that of the GST negative control.

(D) Growth in soft agar. The transfected cells were grown 3 weeks in soft agar, and the colonies were counted and quantitated (top). Representative stained whole dishes are shown (bottom). As expected, GFP-DLC1 suppressed growth in soft agar whereas the biological activity of the talin R8 wt and 2E mutant was similar to that of the GST negative control.

The results in (B)-(D) are represented as means over three experiments ± SD.

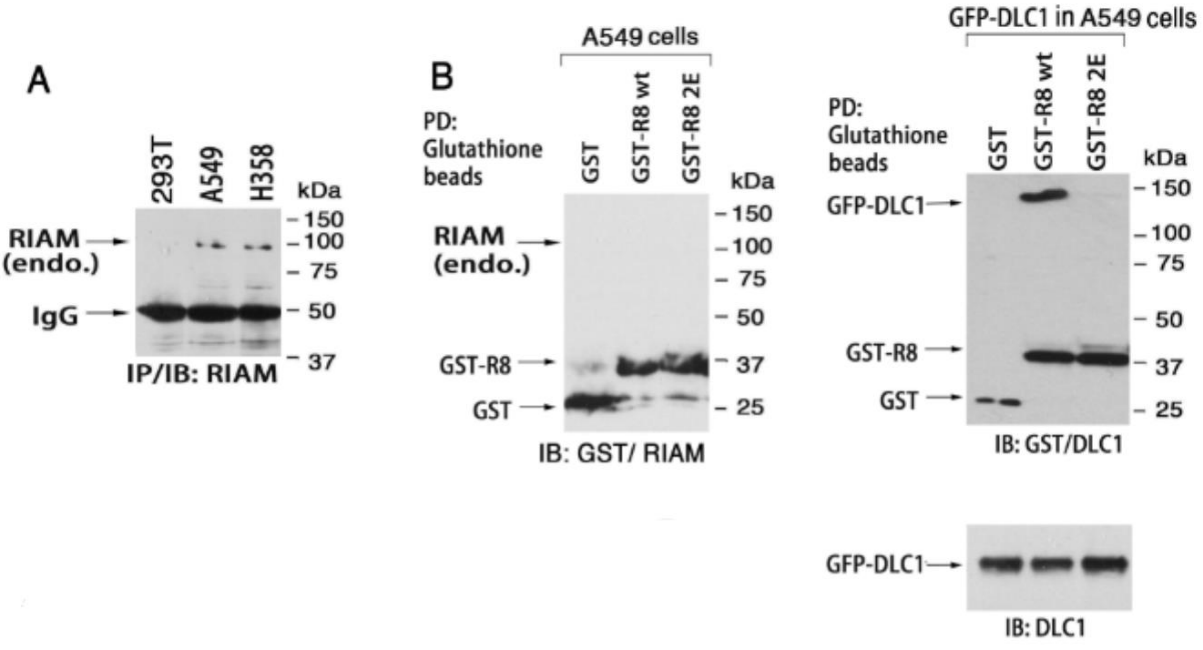

Figure S5, related to figure 6. RIAM is expressed at low levels in A549 cells and does not form a detectable complex with GST-talin R8.

(A) The expression of endogenous RIAM in cell lines. Cell extracts were subjected to immunoprecipitation followed by immunoblotting with anti-RIAM antibody.

(B) A complex between GST-talin R8 and endogenous RIAM is undetectable in A549 cells. A549 cells were co-transfected with GFP-DLC1 and the GST constructs indicated. Cell extracts were subject to a glutathione bead pull down assay followed by immunoblotting with anti-GST and anti-RIAM antibodies on the same membrane (left). No complex between GST-talin R8 and RIAM could be detected. Under the same conditions, wild-type GST-R8 pulled-down the co-transfected GFP-DLC1; the immunoblot with anti-GST and anti-DLC1 on the same membrane is shown (right). The amount of transfected GFP-DLC1 in each sample (loading control) is shown by the anti-DLC1 blot (bottom).

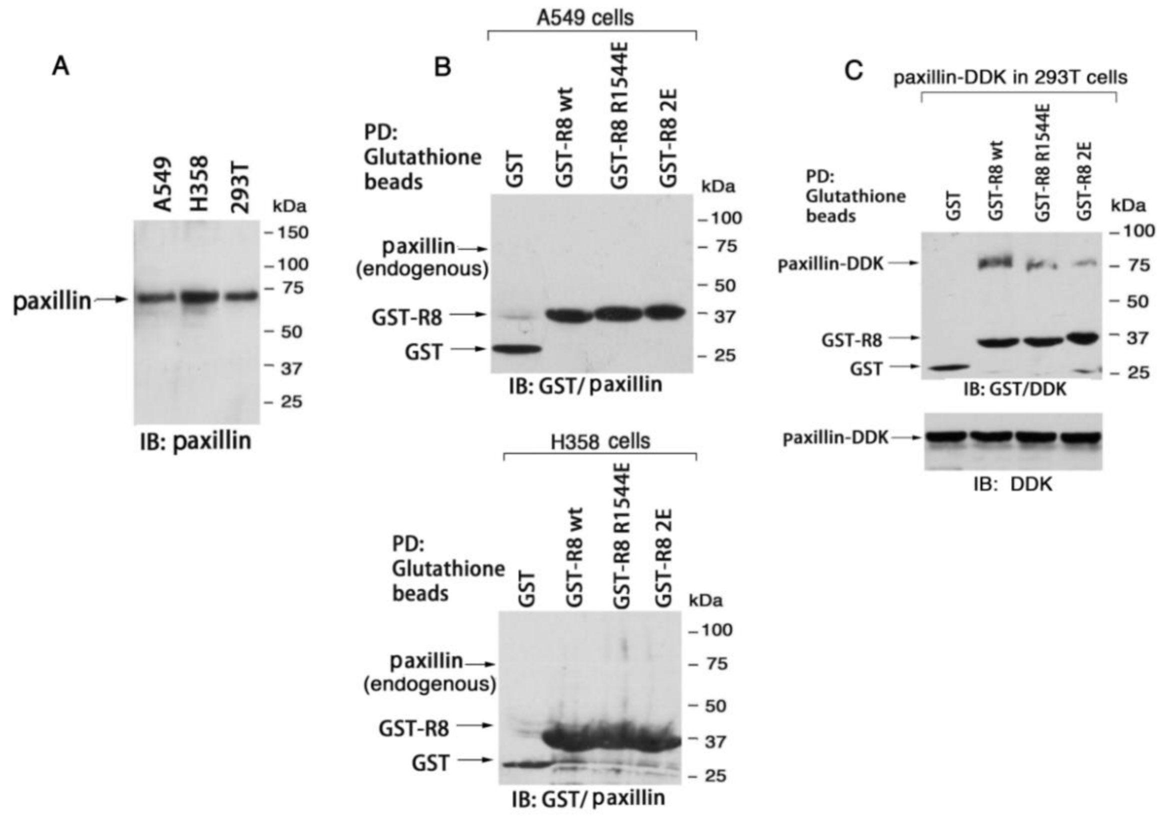

Figure S6, related to figure 5. Binding of GST-talin R8 to endogenous paxillin is undetectable in A549 and H358 cells.

(A) The expression of endogenous paxillin in cell lines. Cell extracts were assayed by immunoblotting with anti-paxillin antibody.

(B) A complex of GST-talin R8 with endogenous paxillin is undetectable in A549 (top) and H358 (bottom) cells. Cells were transfected with the GST constructs indicated, and cell extracts subjected to a glutathione bead pull down assay followed by immunoblotting with anti-GST and anti-paxillin antibodies on the same membrane.

(C) Wild-type GST-talin R8 forms a complex with transfected paxillin in 293T cells. Extracts of 293 cells co-transfected with the GST constructs indicated and paxillin-DDK (OriGene) were subjected to pull-down assays with glutathione beads followed by immunoblotting with anti-GST and anti-DDK antibodies on the same membrane. While GST-talin R8 wt formed a complex with paxillin-DDK, the amount of paxillin complexed to the GST-talin R8 mutants was significantly reduced. The transfected paxillin-DDK in each sample is shown by the anti-DDK blot (bottom) as a loading control.

Supplementary Methods

Peptides and protein preparation – Recombinant wild-type mouse talin1 fragment R7R8 (residues 1357-1653) was previously cloned into pET151/D-TOPO expression vector (Invitrogen), encoding an N-terminal hexa-histidine tag (Gingras et al., 2010). Site directed R7R8 mutants were produced by overlap extension PCR, and subsequent ligation-independent cloning into pOPINB vector (OPPF-UK); constructs were verified by sequencing. Both vectors include N-terminal hexa-histidine tag followed by TEV protease cleavage site.

Proteins were expressed and purified as described previously (Gingras et al., 2010). Briefly, protein was produced in BL21 STAR (DE3) cultured in LB or 2xM9 minimal medium containing 1 g/L of ^{15}N-labelled NH_4Cl. Cells were grown at 37 ^{o}C to an OD_{600} of 0.6, cooled to 18 ^{o}C and induced using 0.5 mM IPTG for 16 hours. Recombinant His-tagged protein was purified by nickel-affinity chromatography following standard protocol. The tag was removed by cleavage with TEV protease, followed by the reverse purification. Protein was further purified using anion exchange chromatography using a 5 ml Hi-Trap QFF column (GE Healthcare).

Paxillin LD1 (^{1}MDDLDALLADLESTTS^{16}) and LD2 (^{141}NLSELDRLLLELNAVQHNPP^{160}) (Mus musculus) were synthesised at the proteomics facility at University of Nottingham; DLC1 TBS peptides (^{467}PELDDILYHVKGMQRIVNQWSEK^{489} and ^{461}ENEDIFPELDDILYHVKGMQRIVNQWSEK^{489}) (Homo sapiens) were synthesised by GL Biochem (Shanghai, China). Peptides were purified to >95% by reverse-phase chromatography and sequence analysed by mass spectrometry.

X-ray crystallography – Diffraction data were collected at beamline i03 (Diamond light source) at a wavelength of 0.97Å. Intensities were integrated and scaled using iMOSFILM and SCALA (Evans, 2006), with 5% of reflections randomly isolated from refinement. The DLC1-R7R8 complex was solved using molecular replacement using the structure of the free R7 domain as a template (PDB: 2X0C) (Gingras et al., 2010). Molecular replacement was performed using PHASER (McCoy et al., 2007) and modelling was performed using COOT 0.8.1 (Emsley and Cowtan, 2004).

Initial electron density maps showed that the position of the R8 domain had changed, and once repositioned, and the R7R8 domain modelled, electron density for the DLC1 peptide was clearly visible, as demonstrated in the simulated annealing composite omit map (Supplementary Figure S1A). Electron density was visible for the entire helix and allowed unambiguous assignment of electron density. Refinement was performed using isotropic B-factors, and at the final stage of refinement employed the use of TLS parameters determined by the TLS motion determination server (Painter and Merritt, 2006), TLS groups included residues 1354-1466 and 1467-1659 of

R7R8 and the entire DLC1 peptide, 467-469. Refinement and validation were performed using PHENIX 1.10 (Adams et al., 2010). Data reduction and refinement statistics are shown in Table 1.

NMR Spectroscopy - Spectra were processed with TopSpin (Bruker) and analysed using CCPN Analysis (Vranken et al., 2005). Resonance assignment was carried out as described previously (Goult et al., 2008). The changes in chemical shift of ^{15}N-talin R8 domain were calculated from ^{1}H,^{15}N-HSQC spectra as $\Delta\delta = \sqrt{(\Delta\delta H^2 + (\Delta\delta N{*}0.15)^2)}$. The cross-peaks corresponding to the bound state were assigned by following chemical shift changes throughout the R8 titration with the increasing amount of peptides.

Dissociation constants were evaluated from the ^{1}H,^{15}N-HSQC chemical shift changes in the titration experiments conducted using 0.1 mM ^{15}N-talin R8 domain. Peptides were added from 5-10 mM stock solutions to generate titration points at 0.1, 0.2, 0.5, 0.75, 1, 2, 4 and 8 peptide:protein ratios. Concentrations of the DLC1 and RIAM peptides were determined from UV absorbance at 280 nm. Concentrations of the paxillin peptide that lacks UV-active aromatic residues was determined by NMR from the comparison of the integral intensities of the well-separated signals of the methyl groups of the paxillin and RIAM peptides following the Eretic2 procedure of TopSpin (Bruker). Concentration of the R8 domain that lacks UV-active aromatic residues was estimated from the comparison with the spectra of the R3 domain that has similar molecular weight and shape using the calibration procedure described in (Wider and Dreier, 2006). Dissociation constants were determined by fitting the chemical shift changes to the equation:

$$\Delta(H,N) = \Delta(H,N)_0 \frac{[P] + [L] + Kd - \sqrt{([P] + [L] + Kd)^2 - 4[P][L]}}{2[P]}$$

where $\Delta(H,N)_0$ is the weighted chemical shift difference at saturation, [P] and [L] are protein and ligand concentrations, respectively. Data were fitted independently for the well-resolved peaks and the average K_d was calculated from 3 different peaks with the lowest standard deviations of the fits. For DLC1 and RIAM only signals in fast exchange regime were selected for the analysis.

DNA Constructs and transfection - The plasmids expressing GFP-DLC1 and GST fusion proteins with talin rod fragments encoding talin amino acids 1288-1646 and 1453-1580 (R8) were described previously (Li et al., 2011). The plasmid encoding 1352-1580 (R7R8) was engineered by PCR, and subcloned into a eukaryotic expression vector, PEBG (Anborgh et al., 1999), using BamHI and NotI sites. The point mutations, K1530E/K1544E, in R8 were made individually and in combination by site-directed mutagenesis using a mutagenesis kit (Agilent). Human Paxillin-DDK (PXN) plasmid was from OriGene.

HEK 293T cells were transfected by lipofectamine 2000 and DLC1-null lung adenocarcinoma cell lines A549 and H358 cells were transfected by lipofectamine 3000 according to manufacturer instructions (Invitrogen). Cells were co-transfected with plasmids expressing GFP-DLC1 or Paxillin-DDK and GST, GST-talin fragments, or vector at a ratio of 1:2.5. Cells were incubated at 37 ^{o}C in a humidified 5% CO_2 atmosphere.

In vivo pull-down assay, co-immunoprecipitation, and immunoblotting - As described previously (Qian et al., 2009), two days after transfection, cell extracts were collected using golden lysis buffer (GLB: 20 mM Tris pH 7.9, 137 mM NaCl, 10% glycerol, 1% Triton, 5 mM EDTA, 1 mM EGTA, 1 mM Na_3VO_4, 10 mM NaF, 1 mM sodium pyrophosphate, 0.5 mM β-glycerophosphate, and protease inhibitor cocktail tablet (Roche)). The cleared supernatants were collected, and the amount of protein estimated by BCA kit (Pierce). Equal amounts of protein from cell extracts were used for pull-down assays by adding 25 µl of glutathione Sepharose-4B slurry (GE Healthcare) and rotating for 3 h at 4 °C. The pellets were washed once with GLB, once with high salt HNTG (20 mM Hepes, 500 mM NaCl, 0.1% Triton-X, 10% glycerol), and twice with low salt HNTG (20 mM Hepes, 150 mM NaCl, 0.1% Triton, 10% glycerol), and incubated with Laemmli sample buffer. After separating the protein samples in SDS-PAGE, the transferred membranes were used for detecting the pull-down proteins by antibody against GST (Santa Cruz), DLC1 (BD Biosciences) and RIAM (Boster Biological Technology Co.) or DDK (OriGene). For co-immunoprecipitation experiments of GFP-DLC1 with endogenous talin, the saved supernatants from each pull-down sample were incubated with anti-talin antibody (Sigma Aldrich). 25 µl of Protein A/G slurry (Pierce) were added to each immune reaction and rotated overnight at 4 °C. The immuno-pellets were washed four times as described for the pull-down assay. Separation of protein samples by SDS-PAGE was followed by immunoblotting using anti-DLC1 or anti-talin antibody. For each blot, horseradish peroxidase-conjugated anti-rabbit or anti-mouse immunoglobulin G (GE Healthcare) was used for the second reaction at 1:10,000 dilution. Immunocomplexes were visualised by enhanced chemiluminescence (ECL), using an ECL kit (GE Healthcare).

G418 colony growth, soft agar growth and cell migration assays - As described previously (Qian et al., 2009), transfected A549 cells were counted, and equal numbers of cells were seeded in triplicate 60 mm dishes (5 x 10^5 per well) overnight, and cultured with RPMI-1640 media containing 0.9 mg/ml G418 (Invitrogen) for 3 weeks. The G418 resistant colonies were stained by 0.5% crystal violet and counted for graph using Prism. For soft agar colony assays, 1 × 10^5 cells were mixed with complete medium containing 0.4% agar (Difco) and grown in 60 mm dishes over a thin layer of 0.6% basal agar. Cells were grown for 2-3 weeks, and colonies were photographed microscopically and quantified by a colony counter. Transwell cell migration assays were performed with 6.5 mm

diameter Falcon cell culture inserts (8 µm pore size; Thermo Fisher) precoated with 0.01% gelatin, in 24 well cell culture plates. Cells were trypsinized and resuspended in serum-free media, then transferred to the upper chamber (5×10^4 cells in 350 µl); 800 µl of media containing 10% FBS were added to the lower chamber. After incubation for 24 h, cells remaining on the upper surface of the filter were removed with a cotton swab; cells that had migrated to the lower surface were fixed, stained with 0.5% crystal violet for 10 min, destained, visualised microscopically, and photographed. The migrated cells were then solubilised overnight with 1% Triton-X-100. The collected lysates were quantified colorometrically in a spectrophotometer using OD_{590nm}.

Ratio imaging - Talin1 and talin2 knock out cells were generated and cultured as described in (Atherton et al., 2015). Transient transfections were performed using Lipofectamine and Plus reagents (Life Technologies) as per the manufacturer's instructions.

Mouse anti-paxillin antibody (clone 349/Paxillin) (BD Transduction Laboratories, Europe) and Goat-anti DLC-1 (sc-32931, Santa Cruz Biotechnology) were diluted (1:500) in 1% Bovine Serum Albumin (BSA) (cat: V9131, Sigma, UK). Dylight 594-conjugated AffiniPure Donkey Anti-Mouse IgG (cat: 715-585-150, Jackson ImmunoResearch, USA) and Dylight 594-conjugated AffiniPure Donkey Anti-Goat IgG (cat: 705-586-147, Jackson ImmunoResearch, USA) were used as a secondary antibodies, diluted in 1% BSA (1:500).

Cells transfected with GFP-talin proteins were incubated overnight on glass bottom dishes (MatTek), fixed with 4% paraformaldehyde and permeabilised with 0.5% Triton X-100 (Sigma). Samples were incubated with the primary antibody for 60 min, and then washed thrice with PBS. Secondary antibody staining followed the same procedure. Fixed samples were imaged using a Delta Vision RT microscope (Applied Precision) equipped with a 60 × /1.42 Plan Apo oil immersion objective (Zeiss). Images were acquired with a CoolSnap HQ camera (Photometrics).

Image analysis was carried out using Fiji ImageJ (version 1.48d) software. All cells analysed had low to intermediate levels of talin. Expression levels were determined by fluorescence intensities measured across a large number of cells exposed to the same amount of fluorescent light. For ratio quantification, GFP-talin and DLC1 or paxillin immunofluorescence images were background subtracted, a region of interest was selected around an individual peripheral adhesion (5 per cell) and the integrated density measured for both channels. Dividing the values from paxillin or DLC1 by talin then produced a ratio.

References

Adams, P.D., Afonine, P.V., Bunkoczi, G., Chen, V.B., Davis, I.W., Echols, N., Headd, J.J., Hung, L.W., Kapral, G.J., Grosse-Kunstleve, R.W., *et al.* (2010). PHENIX: a comprehensive Python-based system for macromolecular structure solution. Acta Crystallogr D *66*, 213-221.

Atherton, P., Stutchbury, B., Wang, D.Y., Jethwa, D., Tsang, R., Meiler-Rodriguez, E., Wang, P., Bate, N., Zent, R., Barsukov, I.L., *et al.* (2015). Vinculin controls talin engagement with the actomyosin machinery. Nat Commun *6*, 10038.
Emsley, P., and Cowtan, K. (2004). Coot: model-building tools for molecular graphics. Acta Crystallogr D *60*, 2126-2132.
Evans, P. (2006). Scaling and assessment of data quality. Acta Crystallogr D *62*, 72-82.
Gingras, A.R., Bate, N., Goult, B.T., Patel, B., Kopp, P.M., Emsley, J., Barsukov, I.L., Roberts, G.C., and Critchley, D.R. (2010). Central region of talin has a unique fold that binds vinculin and actin. J Biol Chem *285*, 29577-29587.
Goult, B.T., Gingras, A.R., Bate, N., Roberts, G.C., Critchley, D.R., and Barsukov, I.L. (2008). NMR assignment of the C-terminal actin-binding domain of talin. Biomol NMR Assign *2*, 17-19.
Li, G., Du, X., Vass, W.C., Papageorge, A.G., Lowy, D.R., and Qian, X. (2011). Full activity of the deleted in liver cancer 1 (DLC1) tumor suppressor depends on an LD-like motif that binds talin and focal adhesion kinase (FAK). Proc Natl Acad Sci U S A *108*, 17129-17134.
McCoy, A.J., Grosse-Kunstleve, R.W., Adams, P.D., Winn, M.D., Storoni, L.C., and Read, R.J. (2007). Phaser crystallographic software. J Appl Crystallogr *40*, 658-674.
Painter, J., and Merritt, E.A. (2006). Optimal description of a protein structure in terms of multiple groups undergoing TLS motion. Acta Crystallogr D Biol Crystallogr *62*, 439-450.
Qian, X., Li, G., Vass, W.C., Papageorge, A., Walker, R.C., Asnaghi, L., Steinbach, P.J., Tosato, G., Hunter, K., and Lowy, D.R. (2009). The Tensin-3 protein, including its SH2 domain, is phosphorylated by Src and contributes to tumorigenesis and metastasis. Cancer Cell *16*, 246-258.
Vranken, W.F., Boucher, W., Stevens, T.J., Fogh, R.H., Pajon, A., Llinas, M., Ulrich, E.L., Markley, J.L., Ionides, J., and Laue, E.D. (2005). The CCPN data model for NMR spectroscopy: development of a software pipeline. Proteins *59*, 687-696.
Wider, G., and Dreier, L. (2006). Measuring protein concentrations by NMR spectroscopy. J Am Chem Soc *128*, 2571-2576.
