# Supplementary material for: LD Motif Recognition by Talin: Structure of the Talin-DLC1 Complex
Source: Structure. 2016 Jul 6;24(7):1130–41. doi: 10.1016/j.str.2016.04.016 (PMC4938799; doi:10.1016/j.str.2016.04.016)
Supplement: Document S2. Article plus Supplemental Information [file mmc2.pdf]

Structure

Article

LD Motif Recognition by Talin: Structure of the Talin-DLC1 Complex

Graphical Abstract

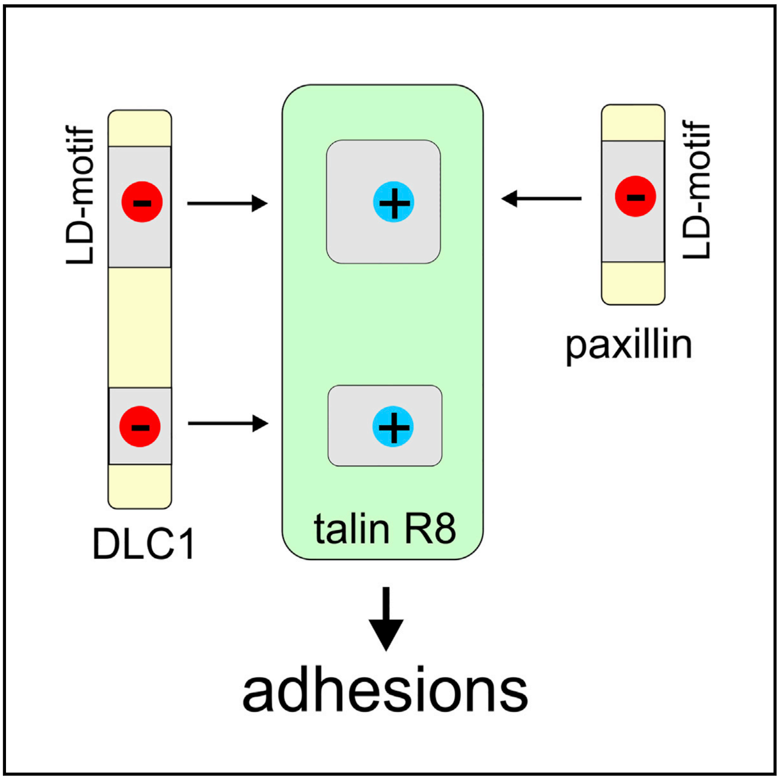

Authors

Thomas Zacharchenko, Xiaolan Qian, Benjamin T. Goult, ..., David R. Critchley, Douglas R. Lowy, Igor L. Barsukov

Correspondence

igb2@liv.ac.uk

In Brief

DLC1 activity depends on binding of its LD motif to talin. Zacharchenko et al. report the structure of the DLC1/talin R8 rod domain complex. They define charge interactions critical for LD-motif recognition by the R8 helical bundle and identify paxillin as a novel talin binding protein.

Highlights

- The DLC1 LD motif forms a helix that binds the talin R8 rod domain
- Talin R8 also binds paxillin LD motifs and recruits paxillin to focal adhesions
- Charge complementarity is key to the interaction between LD motifs and talin R8

Accession Numbers

5FZT

Zacharchenko et al., 2016, Structure *24*, 1130–1141
July 6, 2016 © 2016 The Author(s). Published by Elsevier Ltd.
http://dx.doi.org/10.1016/j.str.2016.04.016

LD Motif Recognition by Talin: Structure of the Talin-DLC1 Complex

Thomas Zacharchenko,[1,6] Xiaolan Qian,[2,6] Benjamin T. Goult,[3] Devina Jethwa,[4] Teresa B. Almeida,[1] Christoph Ballestrem,[4] David R. Critchley,[5] Douglas R. Lowy,[2] and Igor L. Barsukov[1,*]
[1]Institute of Integrative Biology, University of Liverpool, BioSciences Building, Crown Street, Liverpool L69 7ZB, UK
[2]Laboratory of Cellular Oncology, National Cancer Institute, National Institutes of Health, Bethesda, MD 20892, USA
[3]School of Biosciences, University of Kent, Kent, Canterbury, CT2 7NJ, UK
[4]Wellcome Trust Centre for Cell-Matrix Research, University of Manchester, Oxford Road, Manchester M13 9PT, UK
[5]Department of Biochemistry, University of Leicester, Lancaster Road, Leicester LE1 9HN, UK
[6]Co-first author
*Correspondence: igb2@liv.ac.uk
http://dx.doi.org/10.1016/j.str.2016.04.016

SUMMARY

Cell migration requires coordination between integrin-mediated cell adhesion to the extracellular matrix and force applied to adhesion sites. Talin plays a key role in coupling integrin receptors to the actomyosin contractile machinery, while deleted in liver cancer 1 (DLC1) is a Rho GAP that binds talin and regulates Rho, and therefore actomyosin contractility. We show that the LD motif of DLC1 forms a helix that binds to the four-helix bundle of the talin R8 domain in a canonical triple-helix arrangement. We demonstrate that the same R8 surface interacts with the paxillin LD1 and LD2 motifs. We identify key charged residues that stabilize the R8 interactions with LD motifs and demonstrate their importance in vitro and in cells. Our results suggest a network of competitive interactions in adhesion complexes that involve LD motifs, and identify mutations that can be used to analyze the biological roles of specific protein-protein interactions in cell migration.

INTRODUCTION

Integrin-mediated cell adhesion to the extracellular matrix (ECM) involves the assembly of dynamic adhesion complexes and requires the spatial and temporal coordination of signaling and force-transmitting events (Gardel et al., 2010; Wehrle-Haller, 2012). Such complexes form on the cytoplasmic tails of integrin receptors and mature into larger structures called focal adhesions (FA) in response to force exerted by the actomyosin contractile apparatus (Roca-Cusachs et al., 2012). The dimeric adaptor proteins talin1 and talin2 (molecular weight ~270 kDa) play a key role in the assembly of adhesion complexes (Zhang et al., 2008), and talin-null cells cannot adhere or spread on ECM, a phenotype corrected by expression of talin cDNAs (Atherton et al., 2015).

Talin comprises an N-terminal FERM domain (~50 kDa) that binds to and activates integrins, connected to a large flexible rod (~200 kDa) that interacts with multiple ligands, including vinculin and F-actin (Calderwood et al., 2013). Integrin activation is implicated in cancer progression (reviewed in Seguin et al., 2015), and talin overexpression may therefore contribute to cancer metastasis (reviewed in Desiniotis and Kyprianou, 2011). The talin rod constitutes a force-sensing module that regulates the assembly and maturation of adhesion complexes, and is composed of 13 four- and five-helical bundles connected by short linkers, forming an extended flexible chain (Figure 1A) (Goult et al., 2013b). Several rod domains contain cryptic vinculin binding sites (VBSs) that become exposed as the talin domains unfold in response to force, enhancing vinculin binding (del Rio et al., 2009; Fillingham et al., 2005; Papagrigoriou et al., 2004; Yao et al., 2014). Disruption of the talin force-sensing mechanism has strong effects on adhesion assembly, cell polarization, and cell migration (Atherton et al., 2015).

Talin also binds a number of proteins that regulate adhesion dynamics, including the Rap1-GTP interacting protein RIAM (Goult et al., 2013a; Lee et al., 2009), the Rac GEF Tiam1 (Wang et al., 2012), and the Rho GAP DLC1 (Li et al., 2011). Recruitment of Tiam1 and DLC1 to adhesion complexes by talin is likely to have complementary effects, balancing Rac and Rho activity, thus creating a feedback mechanism between actin polymerization, membrane protrusion, assembly of nascent adhesions, actomyosin-driven FA maturation, and FA turnover (Devreotes and Horwitz, 2015; Lawson and Burridge, 2014). The DLC1 binding site in talin has been mapped by deletion analysis to the four-helix R8 domain (Li et al., 2011) that forms a unique protrusion in the C-terminal part of the rod that is otherwise composed of a linear chain of five-helix bundles (Figure 1A) (Gingras et al., 2010). Interestingly, R8 also contains binding sites for RIAM and vinculin, suggesting that the three ligands may compete for binding (Goult et al., 2013b). The talin binding site (TBS) in DLC1 contains an LD-like motif that features in a wide range of other proteins, including the FA protein paxillin (Alam et al., 2014). The TBS in DLC1 interacts with the FA-targeting (FAT) domain of FAK (Li et al., 2011), which also binds the LD motifs in paxillin (Alam et al., 2014). The DLC1 interactions with talin and FAK contribute to the biological activity of DLC1, including its tumor-suppressor activity, establishing the physiological importance of these interactions (Li et al., 2011).

 © 2016 The Author(s). Published by Elsevier Ltd.
This is an open access article under the CC BY license (http://creativecommons.org/licenses/by/4.0/).

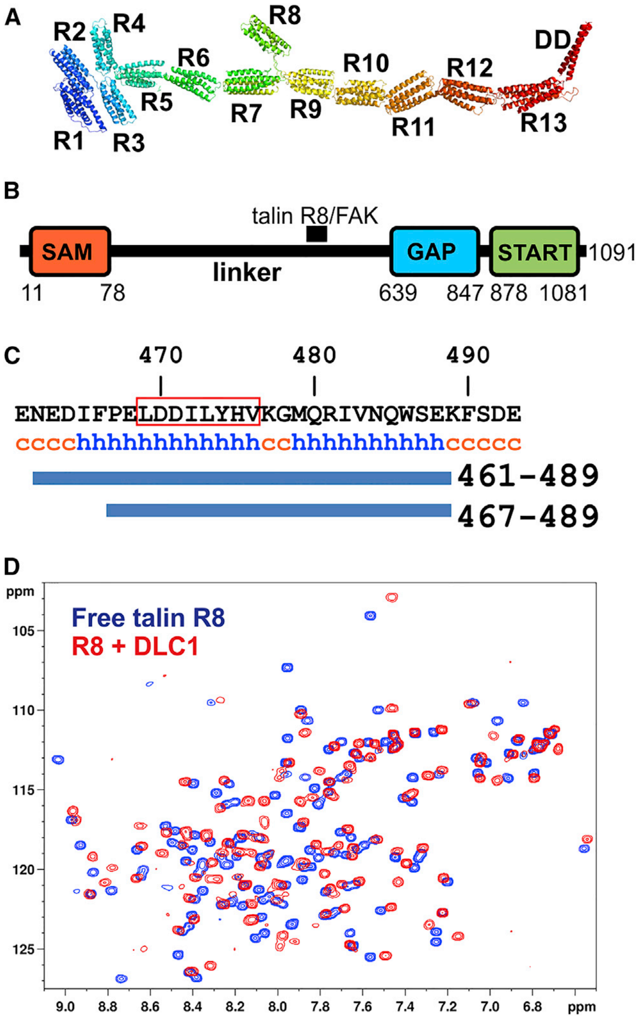

Figure 1. DLC1(467–489) Interacts with the Talin R8 Domain

(A) Model of the talin rod based on the structures of individual domains. Domain R8 interacts with DLC1.

(B) Domain composition of DLC1. The location of the talin binding site (TBS) in the largely unstructured serine-rich linker region is indicated.

(C) Secondary structure prediction for the TBS in DLC1, which includes an LD motif marked by the red box. "h" denotes a region of high helical propensity and "c" a random coil region. Fragments used in this study are indicated by the thick blue lines.

(D) Superposition of the $^{1}H,^{15}N$-HSQC spectra (298 K, 800 MHz) of 100 μM talin R8 domain in the free form (blue) and in the presence of 4-fold excess of DLC1(467–489) (red).

See also Figure S3.

Here we report the crystal structure of the talin R7R8 domains in complex with the TBS of DLC1; the DLC1 LD motif forms a helix that binds to talin R8 in a consensus triple-helix arrangement between the contacting DLC1 and talin helices. We identify the main electrostatic interactions that stabilize the complex and use mutations to demonstrate the importance of the talin/DLC1 interaction in cells. Based on the talin/DLC1 structure, we predicted that talin R8 might also bind paxillin LD motifs; we demonstrate such an interaction by nuclear magnetic resonance (NMR) and glutathione S-transferase (GST) pull-downs, and show that the talin R8 rod domain plays a significant role in recruiting paxillin to FAs. We propose that LD-motif recognition sites in adhesion proteins such as talin and FAK are to a large degree interchangeable, creating a network of competing protein-protein interactions that regulate the properties of adhesion complexes.

RESULTS

Structure of the DLC1/Talin Complex

The TBS in DLC1 has been shown to require an 8-residue peptide ^{469}LDDILYHV^{476} located in the largely unstructured linker region (residues 78–639) between the SAM and GAP domains of DLC1 (Figure 1B) (Li et al., 2011). However, consensus secondary structure prediction using the NPSA server (https://npsa-prabi.ibcp.fr) indicates that the DLC1 peptide is located at the N terminus of a larger region with high helical propensity (residues 465–488, Figure 1C), suggesting that the TBS in DLC1 may extend beyond residues 469–476. To explore this possibility, we used two synthetic DLC1 peptides (residues 461–489 and 467–489) that span the putative helical region. The shorter fragment starts with a proline residue, which usually disrupts helical structure, and is often located at the beginning or end of a helix.

The minimal talin fragment required for interaction with DLC1 (Li et al., 2011) maps to the four-helix bundle R8 domain in the talin rod (Figure 1A) (Gingras et al., 2010; Goult et al., 2013b). Addition of the DLC1(467–489) peptide induced large chemical-shift changes in the heteronuclear single-quantum coherence (HSQC) spectra of ^{15}N-labeled talin R8 (Figure 1D) as did the larger peptide (data not shown), demonstrating the formation of a stable complex. Although the majority of resonances showed significant chemical-shift changes, the overall pattern of cross-peaks was similar to that of free R8, suggesting that the R8 fold does not change upon DLC1 binding.

The shorter DLC1(467–489) peptide was less soluble than the longer fragment, and was therefore less suitable for the NMR titration experiments. However, its lower solubility favored crystallization of a DLC1 peptide/talin complex. For these reasons, we used the longer DLC1 fragment for solution binding studies and the shorter fragment for crystallization experiments. We crystallized a complex of DLC1(467–489) with the talin R7R8 fragment, the structure of which we previously determined in the free form (Gingras et al., 2010), and solved the structure of the complex by molecular replacement (Figure 2A; statistics in Table 1). As in the free form, the R7R8 talin rod fragment adopts a unique fold where the R8 four-helix bundle is inserted into the loop connecting helices α3 and α4 of the R7 five-helix bundle. Individually, the structures of R7 and R8 in the DLC1 complex are nearly identical to that of the free form (root-mean-square deviation [RMSD] 0.35 Å and 1.75 Å, respectively), the main difference being the relative orientation of the two domains (Figure 2B).

The linker region between R7 and R8 is well defined in the crystal structure and shows clear electron density at the 1σ level. It forms a twisted, two-stranded, anti-parallel β sheet stabilized by hydrogen bonds. Each end of the linker has a pair of residues that make close contacts with the helical bundles (Figures 2B and 2C). Despite the different angle between the R7 and R8

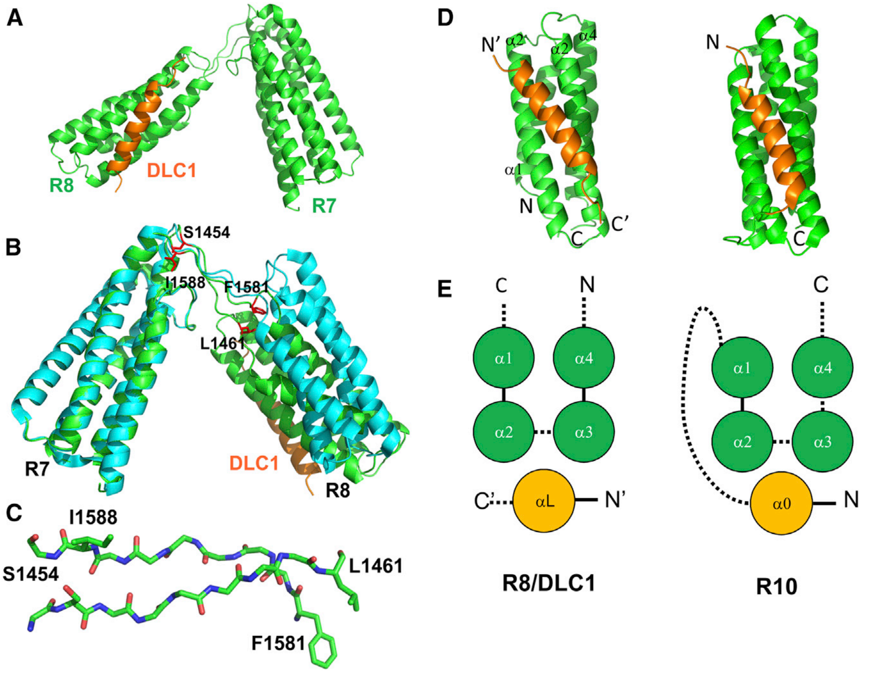

Figure 2. Structure of the Talin/DLC1 Complex

(A) Cartoon representation of the X-ray structure of the talin R7R8 fragment (green) in complex with DLC1(467–489) (orange).

(B) Superposition of the crystal structure of R7R8 in the free form (cyan) and in complex with DLC1(467–489) (green) aligned on the R7 domain. Residues at the ends of the linker regions between R7 and R8 are shown in stick representation (red) and labeled.

(C) Two-stranded anti-parallel twisted β sheet formed in the linker region. Side chains of the residues highlighted in (B) are shown in the stick representation and labeled.

(D) Comparison of the structure of the talin R8/DLC1(467–489) complex (left) and the talin R10 domain (PDB: 2KVP; right). The DLC1 helix and α0 helix of talin R10 are highlighted in orange.

(E) Topology of the talin R8/DLC1(467–489) complex (left) and talin R10 (right).

See also Figure S1.

domains in the complex and free forms, these contacts are maintained in both structures, suggesting that the freedom in domain orientation is mainly defined by the twist and bend of the β-sheet linker. The linker may increase the stability of both domains by bringing together the ends of the helices connected to the linker. In support of the latter possibility, we found a strong effect of surface mutations (R1523E, K1530E, and K1544E) on the solubility of the isolated R8 domain, likely caused by partial unfolding. The same mutations did not affect the fold of the R7R8 double domain (see later).

As expected from sequence analysis and NMR data, the DLC1 peptide forms an α helix that interacts only with the talin R8 domain (Figures 2A and 2D). The peptide is well defined in the structure, with clear electron density at the 1σ level (Figure S1A) and average B-factor values similar to those of the protein (Table 1). Only limited crystal packing contacts were observed between the external surface of the DLC1 helix and the edge of the R7 domain of the neighboring molecule (Figure S1B). The minimal DLC1 binding region (469–476) identified by Li et al. (2011) corresponds only to the N-terminal half of the DLC1 helix, justifying the use of the extended fragment. The helix starts at E468, with the preceding Pro residue having an extended conformation. At the C terminus, the helix ends at W486 with the adjacent SEK sequence (Figure 1C), forming an extended structure.

The DLC1/Talin R8 Complex Resembles a Talin Five-Helix Bundle

The DLC1 helix docks into the hydrophobic groove formed by helices α2 and α3 of talin R8 (Figures 2 and 3), forming a canonical left-handed anti-parallel triple-helix coiled-coil arrangement (Figure S1C) (Lupas and Gruber, 2005). The topology and structure of the DLC1(467–489) complex with talin R8 have a striking resemblance to the five-helix bundles of the talin rod (Figures 2D and 2E). The DLC1 helix is equivalent to the N-terminal helix (designated as α0) of the five-helix bundle that is located at the distant interface between helices α2 and α3 of the four-helix core of the structure in a cross-over arrangement (Goult et al., 2010, 2013b).

As part of the five-helix bundle, the α0 helix makes a set of hydrophobic contacts with the four-helix core. These contacts are mediated by aliphatic side chains located on the hydrophobic face of the amphipathic helix α0, which fits into the hydrophobic pockets at the interface between helices α2 and α3, following the general principle of "knobs-into-holes" packing found in helical bundles (Lupas and Gruber, 2005). The α0, α2, and α3 form a left-handed anti-parallel triple-helix coiled coil that is similar to the triple-helix coiled coil formed by DLC1 with the α2-α3 hairpin of R8 in the complex. The overall structure of the five-helix bundles of the talin rod can thus be classified as conjoined three-/four-stranded coiled coils (Moutevelis and Woolfson, 2009), adding a significant number of new members to this rare fold.

DLC1 Recognition by the Talin R8 Domain

The contacts between DLC1 and R8 are mediated by the hydrophobic side chains of L469, I472, V476, M479, V483, and W486 located on the hydrophobic face of the DLC1 helix (Figure 3D). These residues follow a typical heptad repeat of a coiled coil (Lupas and Gruber, 2005), starting with L468 in position "a" (marked by letters at the top of Figure 3E); the contacting residues occupy positions "a" and "d" of the three sequential repeats. Additional hydrophobic contacts are made by the side chain of L473 in position "e" of the first repeat. At the N-terminal end of the DLC1 helix, corresponding to the LD motif, residues L469, I472, L473, and V476 are embedded between the hydrophobic side chains of L1492 of the R8 α2 helix, and V1540, K1541, and I1543 of the α3 helix in a "knobs-into-holes"

Table 1. Data Collection and Refinement Statistics of the R7R8/DLC1 Complex

Data Collection	
Beamline	I03
Wavelength (Å)	0.97
Resolution range (Å)	55.18–2.1(2.2–2.1)
Space group	$P3_121$
Unit cell	
a, b, c (Å)	73.26, 73.26, 111.82
α, β, γ (°)	90, 90, 120
Unique reflections	20,847
Multiplicity	7.8 (7.6)
Completeness (%)	100
Mean I/σ	10.11 (2.8)
Wilson *B* factor (Å^2)	31.16
R_{merge} (%)	12.8 (65.8)
$CC_{1/2}$	0.998 (0.79)
Refinement	
Unique reflections	19,777
R_{work} (%)	17.66 (20.3)
R_{free} (%)	23.06 (26.4)
No. of atoms	2,635
Macromolecule	2,456
Protein residues	329
RMSD bonds (Å)	0.008
RMSD angles (°)	0.9
Ramachandran favored (%)	98.2
Ramachandran allowed (%)	1.8
Average *B* factors (Å^2)	
R7R8 main chain	34.393
R7R8 side chain	42.621
DLC1 main chain	28.756
DLC1 side chain	38.327
Solvent	39.68

R_{free} is calculated using 5% of data isolated from refinement. Data from highest-resolution shell are in parentheses.

arrangement typical for the coiled-coil packing, creating a small hydrophobic core (Figure 3B). The negatively charged DLC1 residue D470 that is conserved within LD motifs (the "D" residue) makes direct contact with the positively charged side chain of K1544 in R8. The complementary hydrophobic surface of R8, together with the positively charged K1544, creates an LD-recognition box that matches the consensus features of LD binding motifs (Hoellerer et al., 2003) (Figure 3C).

The middle of the α2-α3 binding surface on talin R8 consists of small non-polar side chains that accommodate the hydrophobic residues V476, M479, and V483 in the middle of the DLC1 helix without creating any matching contacts (Figure 3B). This region generally shields the hydrophobic surface of the DLC1 helix from solvent, but is unlikely to make strong contributions to selectivity or affinity. The C-terminal hydrophobic residues V483 and W485 of DLC1 are packed against each other and the side chains of K1510 and V1526 in R8, creating a small hydrophobic cluster that stabilizes the end of the DLC1 helix (Figures 3B–3D).

The polar side chains of Q480 and N484 in DLC1 (positions "e" and "b" of the heptad repeat) make contacts with the matching polar groups of N1534 and N1538 at the edge of the R8 α2-α3 hydrophobic patch, creating a polar ridge (Figure 3B). This ridge is extended by charge contacts between E488 of DLC1, which is wedged between the positively charged groups R1523 and K1530 of R8. These polar residues are not part of the LD motif, but generate DLC1-specific contacts that may contribute to recognition. The interaction between DLC1 E488 and R1523 and K1530 of talin R8 may explain why the DLC1 helix is disrupted at the C terminus: in a continuous helix, E488 would be pointing away from the talin surface.

We tested the role of positively charged residues in talin by selectively reversing the charge of R1523, K1530, and K1544 (Figures 3B and 4A). Surprisingly, when these mutations were introduced into the isolated R8 domain, a large fraction of the protein was found in inclusion bodies and the soluble fraction contained partially degraded protein. These observations suggest that although the mutations were at solvent-exposed positions, the R8 fold was destabilized. In contrast, the talin R7R8 fragment bearing the same mutations was soluble and stable. Similarity of the NMR spectra of the wild-type and mutated R7R8 demonstrate that the protein fold was not affected.

Single-residue mutations in talin R8 had variable effects on DLC1 binding to talin. The spectral changes for the R1523E talin R7R8 mutant were the closest to those of wild-type, with large shifts and broadening of the signals indicating minimal effects on DLC1(461–489) binding (Figure S2A). Somewhat reduced shift changes and significantly less broadening was observed for the K1544E mutation (Figure 4C), and very limited shift changes with no additional broadening were observed for the K1530E mutant (Figure 4D). From these results, we conclude that K1530 makes the largest contribution to the interaction with DLC1. The contribution of K1544 is significant, but smaller, while the contribution of R1523 is negligible. However, none of the single mutations completely abolished the interaction with DLC1. To enhance the effects of the mutations, we generated the K1530E/K1544E double mutant; this 2E R7R8 double mutant showed negligible chemical-shift changes on addition of DLC1 (Figure 4E), effectively disrupting the interaction between talin R8 and DLC1.

To validate the ion pairing between D470 and E488 of DLC1, and K1530 and K1544 of talin R7R8, we introduced charge-reversal mutations D470K/E488K in DLC1, complementary to K1530E/K1544E of talin. The addition of the double D470K/E488K DLC1 mutant to the K1530E/K1544E talin R7R8 induced significant chemical-shift changes (Figure 4F). These changes were not as large as those observed with the wild-type proteins, but were comparable with changes observed with the K1530E mutant. The D470K/E488K DLC1 mutant also showed some interaction with the wild-type R7R8, although not as strong as with the wild-type DLC1 (Figure S2B). The incomplete recovery of the interaction and residual binding of the mutated DLC1 may reflect the ability of the peptide to adopt a slightly different conformation in the complex due to its small size and flexibility. Although further optimization will be required to enhance the interaction between the DLC1/talin R8 charge-reversal mutants,

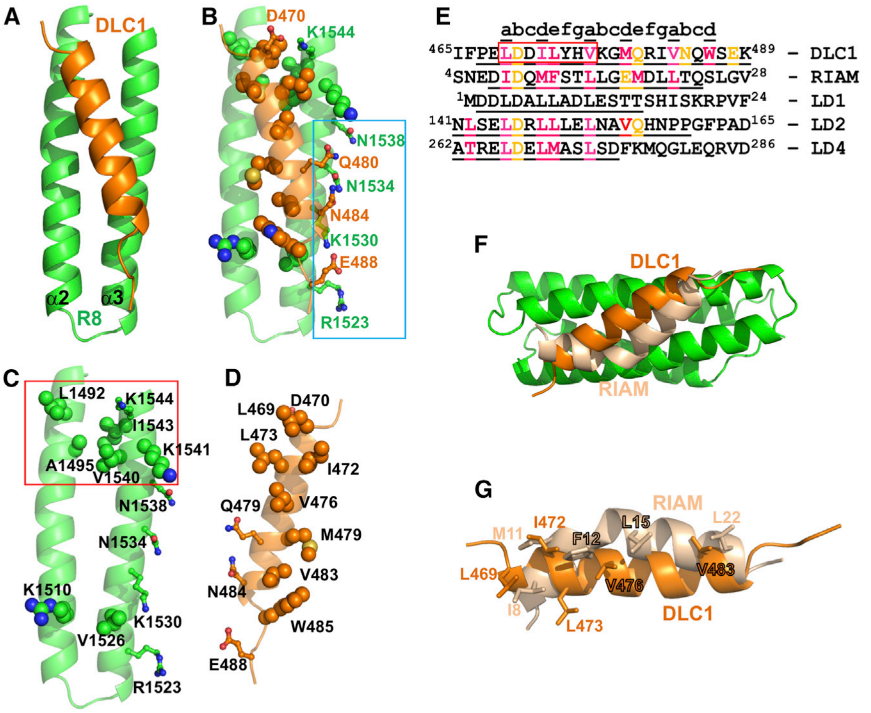

Figure 3. Recognition of the DLC1(467–489) Helix by the Talin R8 Domain

(A) Position of the DLC1(467–489) helix (orange) relative to the α2 and α3 helices of talin R8 (green).

(B) DLC1 and talin residues that make contacts in the complex. Side chains of the residues involved in hydrophobic interactions are shown as balls; charged and hydrophilic interactions are shown as balls and sticks. Blue rectangle identifies the "polar ridge" of the complex.

(C) DLC1-interacting residues on the talin surface. LD-recognition box is marked by a red rectangle.

(D) Talin-interacting residues on the surface of DLC1 helix. The helix is rotated by 180° around the vertical axis relative to the orientation in (B).

(E) Sequence alignment of DLC1 with RIAM TBS and paxillin LD domains. Peptide fragments used to solve the structures of the complexes are underlined. Residues involved in the interactions with the corresponding proteins are highlighted in magenta (hydrophobic interactions) and orange (charged and hydrophilic interactions). Red box indicates the DLC1 LD-motif identified from sequence comparison. For paxillin LD1 the underlined region corresponds to the LD motif. Positions of the coiled-coil heptad repeat are shown above the sequences. The underlined positions "a" and "d" correspond to the interacting hydrophobic residues in coiled coils.

(F) Comparison of the positions of DLC1 and RIAM helices in the complexes with the talin R8 domain.

(G) Locations of the hydrophobic residues on the surfaces of the DLC1 and RIAM helices involved in the interaction with talin R8. The helices are rotated by 180° around the horizontal axis relative to the orientation in (F).

See also Figure S1.

the results support the roles of the charged residues in DLC1 recognition by talin.

Comparison of DLC1, RIAM, and Paxillin Complexes

The talin binding LD motif of DLC1 interacts with the LD binding FAT domain of FAK and was initially identified through its homology with paxillin LD motifs (Li et al., 2011). From the sequence homology and structural similarity, we predicted that paxillin LD motifs should also interact with the talin R8 domain. Indeed, we observed large chemical-shift changes in the ^{1}H,^{15}N-HSQC spectra of talin R8 and R7R8 on addition of paxillin LD1 (Figures 5A and S2C) and LD2 peptides (data not shown). The amplitudes of the chemical-shift changes were comparable with those induced by DLC1 (compare with Figure 1D), although a smaller number of resonances were affected. The chemical-shift changes map predominantly to the LD motif binding region of the talin R8 domain (Figure 5B). No chemical-shift changes were detected on the interfaces formed by other R8 helices, demonstrating that R8 has only a single LD motif binding site unlike the FAT domain of FAK, which has two (Figure 5B) (Hayashi et al., 2002; Hoellerer et al., 2003).

Overall, the topology of the R8/DLC1 and FAK/paxillin complexes is similar, and binding is mediated by similar residues (Figures 3E and 5B), suggesting that the paxillin LD motif interacts with the LD-recognition box in talin R8. In this orientation only a single ion pair between K1544 of talin R8 and the D residue of the paxillin LD motif is expected to form, potentially making the contribution from this contact more prominent. Consistent with this prediction, we detected only minor chemical-shift changes in the K1544E talin R7R8 mutant on addition of paxillin LD1 (Figure 5C).

Using an LD motif deletion mutant of DLC1, we previously demonstrated that the DLC1/talin interaction contributes to DLC1-adhesion targeting (Li et al., 2011). To assess whether the interaction with talin R8 has similar effect on paxillin localization, we compared talin/paxillin and talin/DLC1 ratios in talin1 and talin2 knockout (TKO) cells (Atherton et al., 2015) transfected either with wild-type talin or a talin mutant lacking the R8 domain (talΔR8). The relative abundance of both DLC1 and paxillin in adhesions was significantly and comparably reduced in cells expressing talΔR8 (Figure 5D). Reduced DLC1 localization was analogous to what we had seen earlier with the DLC1 mutant (Li et al., 2011), providing independent evidence that talin R8 is the interaction site for DLC1, thus validating our approach. The reduced localization of paxillin in FA provides the first evidence that talin directly contributes to paxillin recruitment to FA.

Besides DLC1 and paxillin, the R8 domain also binds RIAM (Goult et al., 2013b). The recently reported structure of the R8/RIAM complex (PDB: 4W8P) (Chang et al., 2014) shows that, similar to DLC1, RIAM forms a helix that fits into the hydrophobic groove of the α2 and α3 helices of talin R8 (Figure 3F; Chang et al., 2014). Although not identified as an LD motif, the sequence of RIAM has a characteristic distribution of negatively charged and hydrophobic residues (Figure 3E) that explains the interaction with the LD-recognition surface of R8. In support for the similarity of DLC1 and RIAM recognition by R8, we observed a

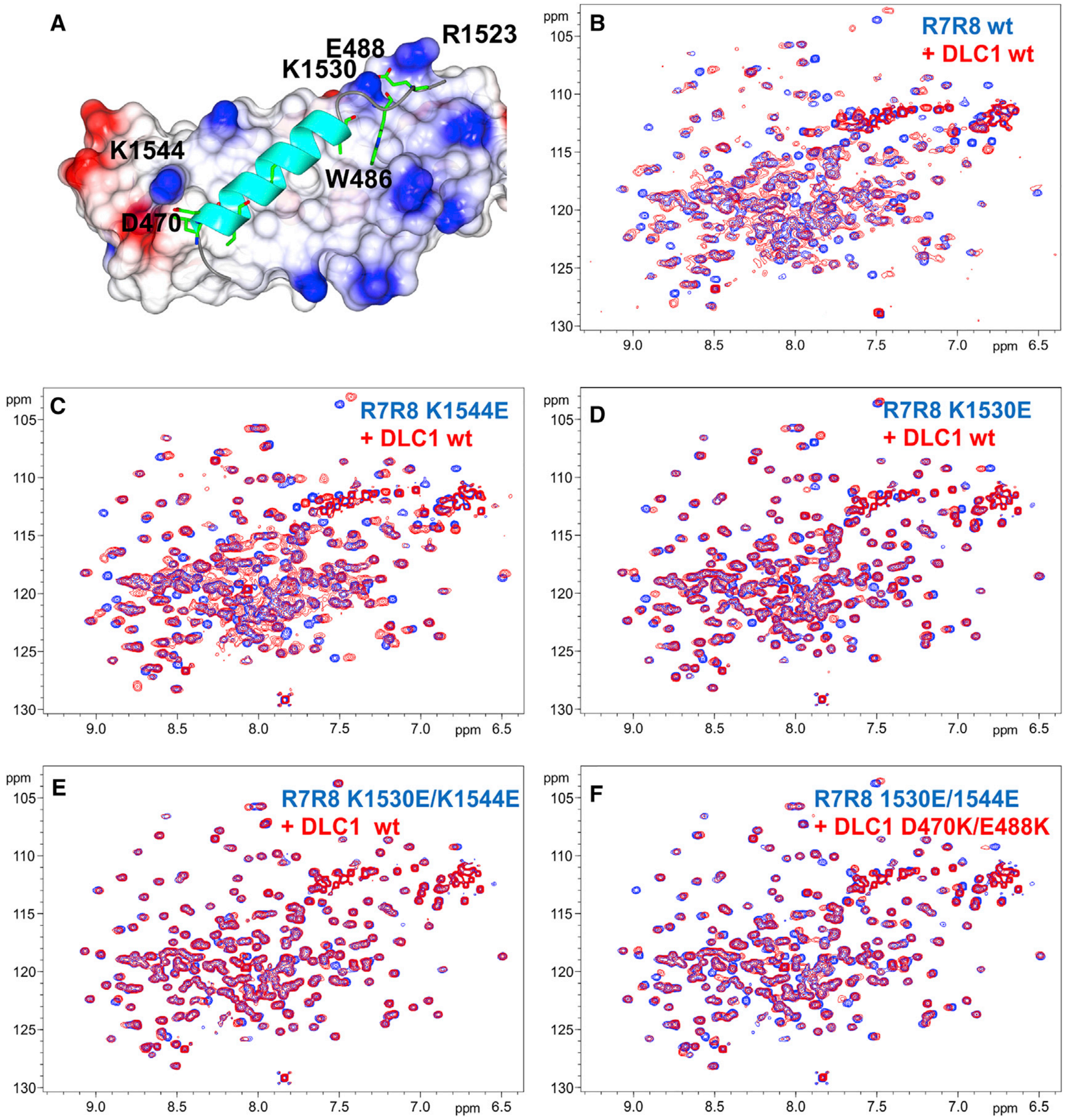

Figure 4. Interactions of Charge-Reversal Mutations of Talin R7R8 and DLC1(461–489)

(A) Location of the mutated residues in the structure of talin R8/DLC1(467–489) complex.

(B–F) Superposition of the HSQC spectra of 0.2 mM talin R7R8 free (blue) and in the presence of 0.8 mM DLC1(461–489) (red). Mutations are marked on the spectra. wt, wild-type form of the protein.

See also Figure S2.

strong reduction in RIAM binding affinity for the R1530E/K1544E mutant (Figures S2E and S2F).

Interestingly, in the R8/RIAM complex (Chang et al., 2014) the RIAM helix has an unusual kink, which causes its displacement relative to DLC1 (Figure 3F). However, the critical hydrophobic side chains that make contacts with the surface of talin R8 are located in similar positions, and make contacts with similar residues on R8, particularly at the N- and C-terminal ends of the helices (Figure 3G). These residues occupy equivalent positions in the sequences of the two proteins, showing that the DLC1 and RIAM helices are generally in register relative to each other (Figure 3E).

The kink in the RIAM helix appears to be forced by the hydrophobic contacts of the aromatic ring of F12, which is inserted between helices α2 and α3 of talin R8. In DLC1, the equivalent L473 occupies a peripheral position and is partly exposed to solvent. The helical kink is energetically unfavorable, but may be partially compensated by the hydrogen bond involving RIAM S13, as suggested by Chang et al. (2014). Significantly, no kink is present in the RIAM helix in complex with vinculin determined by X-ray crystallography (Goult et al., 2013b), nor with the talin F3 domain determined by NMR (Yang et al., 2014). These arguments support an induced kink model, rather than a stable kinked helix model proposed by Chang et al. (2014). Additional contributions

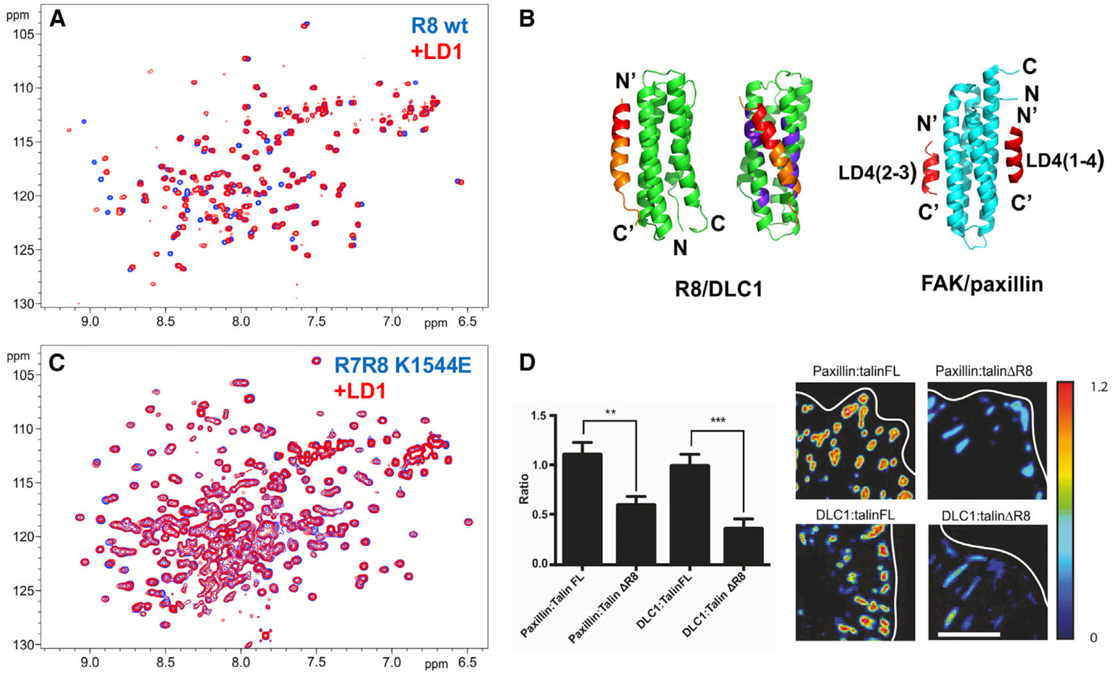

Figure 5. Interaction of Paxillin LD Motifs with Talin R8

(A) Superposition of the HSQC spectra of 0.1 mM talin R8 free (red) and in the presence of 0.4 mM paxillin LD1 (blue).

(B) Comparison of structures of talin R8/DLC1 and FAK/paxillin complexes. From left to right: side view of the R8/DLC1 complex—the DLC1 helix is in orange with the LD motif highlighted in red; front view of the R8/DLC1 complex—largest chemical-shift perturbations caused by LD1 binding are highlighted in purple; structure of the FAK complex with LD2 bound to the 2–3 site (helices α2 and α3) and LD4 bound to the 1–4 site (helices α1 and α4) (PDB: 1OW7).

(C) Superposition of the HSQC spectra of 0.2 mM talin R7R8 K1544E mutant free (red) and in the presence of 0.8 mM paxillin LD1 (blue).

(D) Ratio imaging was used to determine the proportion of endogenous paxillin and DLC1 present at FA in TKOs expressing either talin FL or talin ΔR8. Quantitative analysis shows that both paxillin and DLC1 are markedly reduced in adhesions when talin R8 is deleted (n = 20 cells from three independent experiments). Error bars are ± SEM. **p < 0.01, ***p < 0.001 (ANOVA). White line indicates cell margin. Scale bar, 10 μm.

See also Figures S2 and S3.

to the kink in the RIAM helix may be due to crystal packing (Figure S1D).

Changes in the NMR spectra of R8 on ligand addition suggest different affinities for the interactions between talin and DLC1, RIAM, and paxillin. The strongest effects on the spectra were observed for RIAM, where many signals shifted and broadened significantly at R8/peptide ratio as low as 1:0.1. For DLC1 similar broadening and shifts were observed, but required a higher ratio of 1:0.5, while for paxillin only chemical-shift changes were detected. For each peptide, the chemical-shift changes of the signals that showed only limited broadening throughout the titration (corresponding to a fast exchange regime) could be successfully fitted to the theoretical binding curves, with similar dissociation constants (Figure S3). In agreement with the qualitative analysis, the K_D values determined by fitting were 48, 3.5, and 168 μM for DLC1, RIAM, and paxillin, respectively. Overall, the measured K_D values are within the range of the low to high-micromolar values reported for biologically relevant LD-motif interactions (Alam et al., 2014), and the value for RIAM is in excellent agreement with that reported earlier (Chang et al., 2014). The high affinity of talin R8 for RIAM likely reflects the larger contribution of hydrophobic side chains to binding, while the lower affinity for paxillin correlates with the smaller binding region.

Biological Implications for DLC1-Talin Interaction from Mutational Analysis

We reported previously that wild-type talin R8 is sufficient to form a complex with full-length DLC1 in cells (Li et al., 2011). To evaluate the effects of the single K1530E and K1544E and double K1530E/K1544E (2E) R8 mutants on the complex formation in vivo, we engineered GST-tagged R8 constructs into isogenic mammalian expression plasmids and co-transfected them with GFP-DLC1 into HEK 293T cells. Complex formation was determined by a GST pull-down assay. Consistent with the NMR results, the talin R8 K1530E mutation caused a greater reduction in DLC1 binding than K1544E, while the 2E double mutant reduced binding to a greater extent than either single mutant (Figure S2D).

We next compared the ability of the wild-type talin R8 and mutant constructs to compete with binding of endogenous talin to GFP-DLC1 in cells, to see whether the GFP-DLC1-dependent biological effects require the interaction with talin R8. For this experiment we used three pairs of GST-tagged talin constructs that each contained R8; (1) the wild-type talin R8 and 2E constructs described above (encoding amino acids 1,453–1,580), (2) talin R7R8 and equivalent 2E constructs (encoding amino acids 1,352–1,580), and (3) wild-type and 2E talin constructs spanning residues 1,288–1,646 that were used

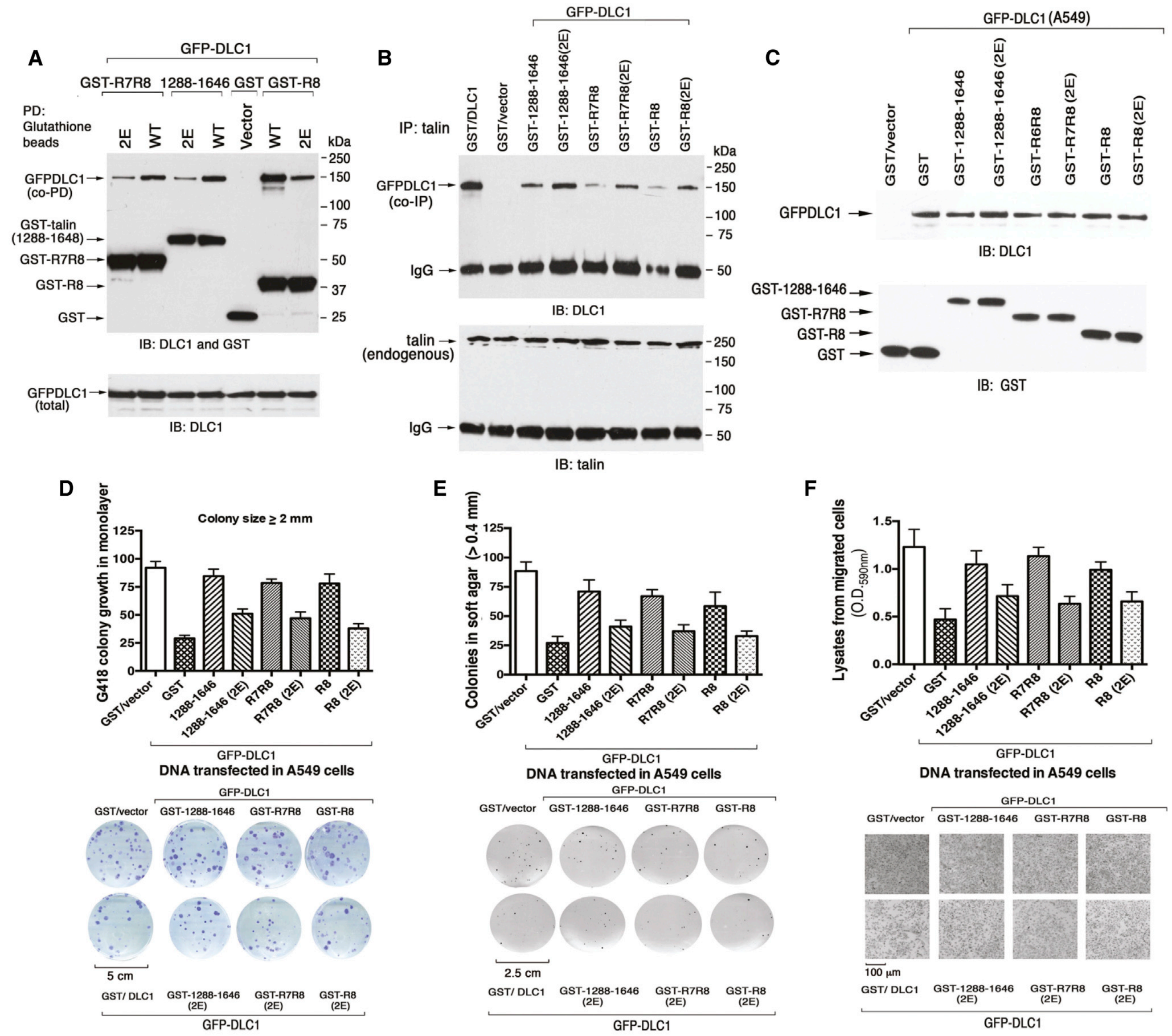

Figure 6. Talin R8 Mutations Disrupt the Interaction with DLC1 and Affect Its Biological Activity

(A) Wild-type GST-talin fragments pull down more DLC1 than the 2E mutants. Extracts of HEK293T cells transfected with GFP-DLC1 and GST-talin constructs were subjected to pull-down assays with glutathione beads followed by immunoblotting with anti-GST and anti-DLC1 on the same membrane (top). The transfected GFP-DLC1 in each sample is shown by the anti-DLC1 blot (bottom) as a loading control.

(B) Wild-type GST-talin fragments compete efficiently with endogenous talin to form a complex with DLC1. The supernatants collected after pull-down assay from (A) were reused for co-immunoprecipitation with an anti-talin antibody and blotted with anti-DLC1 (top). A small aliquot from each lane was blotted for endogenous talin as a loading control (bottom).

(C) Co-expression of GST or GST-talin fragments (wild-type or 2E mutant) with GFP DLC1 in A549 cells. Six days after transfection, A549 cell lysates were blotted with anti-DLC1 (top) and anti-GST (bottom) to conform equal protein expression.

(D) G418 colony growth assay. Transfected A549 cells were cultured in G418 for 3 weeks, and colonies counted and quantitated (top). Representative stained colonies are shown (bottom).

(E) Growth in soft agar. Transfected A549 cells were grown for 3 weeks in soft agar, and colonies counted and quantitated (top). Representative stained whole dishes are shown (bottom).

(F) Transwell cell migration assay. Lysates from migrated cells were quantitated (top), and representative microscopic images of the migrated cells are shown (bottom).

The results in (D–F) are represented as means over three experiments ± SD. See also Figures S4–S6.

previously (Li et al., 2011). GST served as negative control in the assay. We first confirmed that complex formation with GFP-DLC1 as determined by GST pull-downs was greater for each wild-type talin fragment than for the respective 2E mutant (Figure 6A). The wild-type versions of each talin construct should therefore compete with endogenous talin for binding to

GFP-DLC1 more effectively than the 2E mutant. Talin was immunoprecipitated from the supernatants of the GST pull-downs and blotted for GFP-DLC1 to evaluate this; co-expression of GST with DLC1 or with vector served, respectively, as a positive and negative control (Figure 6B). Substantially less GFP-DLC1 co-immunoprecipitated with talin in cells co-transfected with constructs containing wild-type R8 versus the 2E mutants (Figure 6B). We conclude that each wild-type GST-talin polypeptide inhibits binding of GFP-DLC1 to endogenous talin more effectively than the respective 2E mutant.

To assess the biological effects of inhibiting the interaction between endogenous talin and GFP-DLC1, we tested the ability of each talin wild-type and 2E mutant pair to antagonize the activity of co-transfected GFP-DLC1 in the A549 human non-small cell lung cancer line. Equivalent expression levels of each talin construct were confirmed by western blotting (Figure 6C). We used three different bio-assays (for details see Supplemental Experimental Procedures): monolayer colony growth (Figure 6D), growth in soft agar (Figure 6E), and transwell cell migration (Figure 6F). In the absence of any co-transfected talin fragment, GFP-DLC1 was inhibitory in all three assays, while the GST-R8 talin construct (wild-type or 2E mutant) by itself had no detectable biological activity, as its effects were similar to that of the GST negative control (Figure S4). However, each wild-type talin polypeptide attenuated the inhibitory activity of GFP-DLC1 in all three bio-assays, consistent with its efficient displacement of endogenous talin from GFP-DLC1. By contrast, each 2E mutant had only a marginal effect on the inhibitory activities of GFP-DLC1. The results clearly demonstrate that the biological activity of DLC1 is associated with its interaction with talin and confirm the importance of the talin R8 residues K1530 and K1544 to the interaction.

However, as talin R8 interacts with RIAM (Goult et al., 2013b) and paxillin (shown here) in addition to DLC1, we used several approaches to evaluate whether binding of talin R8 to endogenous RIAM or paxillin might have contributed to the observed results. For RIAM, the level of expression in the cell lines used here varied from very low to undetectable. To detect RIAM protein in any of the cell extracts, we had to use an anti-RIAM immunoprecipitation step followed by anti-RIAM immunoblotting. Using these conditions, endogenous RIAM was detected in A549 and H358 cells, but not in 293T cells (Figure S5A). In A549 cell extracts, which contain endogenous RIAM, anti-RIAM immunoblotting did not detect a GST-R8 complex (Figure S5B, left), whereas the wild-type GST-R8, but not the 2E mutant, did bind GFP-DLC1 under the same conditions (Figure S5B, right). The failure to detect an R8/RIAM complex despite the higher affinity of R8 for RIAM versus DLC1 suggests that the biological effects induced by GST-R8 are unlikely to be mediated via RIAM.

To investigate whether the biological effects of GST-R8 might be partly mediated via paxillin, we first confirmed that endogenous paxillin is expressed in cell lines A549, H358, and 293T (Figure S6A). However, the levels of endogenous paxillin in A549 and H358 cells, in combination with its relatively low affinity for DLC1, were insufficient to detect binding to GST-R8 using the pull-down assay (Figure S6B top and bottom, respectively). As a positive control, HEK293 cells were co-transfected with a paxillin-DDK construct (OriGene) and GST-R8 (wild-type, R1544E and 2E mutants), followed by a GST pull-down assay. Under these conditions, wild-type GST-R8 did bind paxillin-DDK, and did so more efficiently than 2E GST-R8 talin mutant (Figure S6C).

Taking these data together, we conclude that the ability of wild-type GST-talin R8 to inhibit growth and migration in A549 cells is largely attributable to its interaction with DLC1, as no effect was observed in the absence of DLC1, and binding to endogenous RIAM and paxillin in cell extracts was undetectable under conditions associated with a strong DLC1 interaction.

DISCUSSION

The interaction between talin and DLC1 plays a key role in recruiting DLC1 to FAs and contributes to the tumor-suppressor activity of DLC1 (Li et al., 2011). Although deletion analysis has been successfully used to identify regions that are critical for talin interaction with DLC1 (Li et al., 2011), the exact location of the binding sites and the mechanism of the interaction have remained unknown. Here, we refine the boundaries of the TBS in DLC1 and report the crystal structure of this region in complex with the talin R7R8 rod domains. Analysis of the structure identifies the general features of the DLC1 binding site in the talin R8 four-helix bundle and the specific residues involved. Thus, a talin R8 K1530E/K1544E double mutant markedly reduced binding to DLC1 peptides in vitro, and to full-length DLC1 in cells, compromising the ability of GST-talin R8 constructs to displace DLC1 from endogenous talin and thereby to attenuate the tumor-suppressor activity of DLC1. Sequence similarity between the TBS in DLC1 and paxillin LD motifs suggested a possible interaction between talin and paxillin, and we have confirmed this novel interaction by NMR and have shown that it is an important factor in determining paxillin levels in FAs. Taken together, our results explain how talin R8 recognizes LD motifs in both DLC1 and paxillin, and suggest that talin forms part of an LD-motif-based network of interacting proteins that contribute to the assembly and regulation of adhesion complexes.

Our structure of the talin R7R8/DLC1 complex demonstrates that the TBS in DLC1 forms a helix that packs against the two adjacent α2 and α3 helices of the talin R8 four-helix bundle in a consensus left-handed triple-helix coiled-coil arrangement. The DLC1 binding site in talin is fully accessible to solvent, and the conformation of the R8 domain does not change on binding. The resulting five-helix coiled-coil complex can be classified as a hybrid conjoined three-/four-stranded coiled coil (Moutevelis and Woolfson, 2009). A similar structure is formed in the talin R8/RIAM (Chang et al., 2014) and paxillin/FAK (Hoellerer et al., 2003) complexes. Although classified as a rare fold (Moutevelis and Woolfson, 2009), the three-/four-stranded coiled coil is likely to be a relatively common topology for complexes between four-helix bundles and isolated helices, as it minimizes the rearrangement of the four-helix core.

Recognition that the interaction between DLC1 and talin R8 involves coiled-coil packing allowed us to analyze the interaction, using well-established rules for coiled-coil structures. The TBS in DLC1 contains a typical heptad repeat identified in left-handed coiled coils (Lupas and Gruber, 2005) (Figure 3) that creates a hydrophobic interaction surface. Flanking this region are polar residues that contact complementary polar residues in talin R8. We identified three regions on the talin R8 surface that aid

recognition of the DLC1 helix: (1) an LD-recognition box consisting of a hydrophobic cluster with an embedded positive charged amino acid that matches the consensus LD motif, (2) a polar ridge that generates a network of polar contacts and hydrogen bonds between DLC1 and R8, and (3) a small hydrophobic patch that contacts the C-terminal hydrophobic residues of the DLC1 helix. In addition, the R8 binding surface lacks any charged or large polar residues along the whole interface between the α2 and α3 helices, thus avoiding any unfavorable contacts with the hydrophobic residues in the middle of the DLC1 helix. Together, these features create a complementary surface that can accommodate the entire length of the DLC1 TBS helix (Figure 3).

Among the contacts identified between DLC1 and talin R8, charge complementarity within the polar ridge (Figure 3) is likely to define ligand selectivity. We confirmed this prediction by reversing the charges of K1530 and K1544 at opposite ends of the binding region in R8. While double charge reversal completely abolished DLC1 binding, single charge reversals had only a partial effect, demonstrating that both interactions contribute to ligand recognition. Paxillin LD motifs form significantly shorter helices that correspond to the N-terminal half of the DLC1 helix, and interact only with the LD-recognition box. In this case charge reversal of K1544 in the LD-recognition box of R8 (Figure 3C) had a much stronger effect on the interaction with paxillin, practically abolishing binding. This observation highlights charge complementarity as a general feature of LD motif recognition, with additional contributions outside the LD box fine-tuning the interactions with specific ligands.

Our results further support the important contributions of weak interactions to the adhesion mechanisms. Despite the relatively low affinities of DLC1 and paxillin for talin R8 (K_D of 48 and 168 μM, respectively), these interactions can be detected in cells, and their disruption strongly reduces the abundance of DLC1 and paxillin in FAs (Figure 5D). For DLC1 this affects adhesion-dependent colony growth and migration, although the biological role of the talin-paxillin interaction is currently unclear and will need further investigation. Large differences in the dissociation constants of DLC1, RIAM, and paxillin interactions with talin R8 are in line with the low- to high-micromolar range of constants determined for other LD motif interactions (Alam et al., 2014). These interactions are likely to be enhanced through the high concentration of the binding sites within adhesion complexes.

Although not previously identified as an LD motif, the N-terminal part of the TBS in RIAM shows a pattern similar to that of DLC1, with hydrophobic and charged residues that fit the LD-recognition box in talin R8 (Figure 3). DLC1 also binds to the FAK FAT domain, a recognized partner for paxillin LD motifs, and R8 itself interacts with paxillin. Extending this set of interactions, other LD motif binding proteins that have four-helix bundle structures, such as PYK2 (Alam et al., 2014), may also interact with DLC1 and RIAM. In turn, LD motifs of other proteins, including members of the paxillin family, such as leupaxin and Hic-5, may interact with talin. The combination of an LD-like helix and a four-helix bundle containing an LD-recognition box may be a common feature among interacting adhesion proteins serving alongside other interacting pairs such as SH3 domain/polyproline sequences. The critical contribution of charged residues to recognition of the LD motif and additional interactions outside the LD motif can be used to selectively modulate the binding of specific ligands, as we demonstrated for DLC1, paxillin, and RIAM using charge-reversal mutations.

Comprehensive analysis of talin has revealed multiple ligand binding sites in the 13 talin rod domains, often arranged in complex overlapping patterns (Goult et al., 2013b). There are 11 VBSs in the talin rod, and the talin/vinculin interaction plays a key role in stabilizing FAs (Carisey et al., 2013). There are five putative RIAM binding sites in talin (four in the rod) that have the potential to regulate the initiation of adhesion complex assembly (Goult et al., 2013b; Yang et al., 2014). In addition, we now identify a paxillin binding site in the talin rod, and more talin interactions may be discovered. In turn, RIAM itself has two TBSs that can also bind vinculin (Goult et al., 2013b), and paxillin has five LD motifs, several of which interact with vinculin and FAK (Hoellerer et al., 2003). A direct link between talin and FAK has also been reported (Lawson et al., 2012; Lawson and Schlaepfer, 2012), although molecular details of this interaction are missing. DLC1 has at least one binding site that interacts with talin and FAK in a similar way. All these interactions create a complex network at the core of adhesion complexes, where mechanosensing molecules such as talin and vinculin link to each other and to signaling molecules such as FAK and DLC1, either directly or indirectly, through adaptor proteins such as RIAM and paxillin.

Strikingly, all talin rod five-helix bundles, except the C-terminal R13 actin-binding domain (Gingras et al., 2008; Goult et al., 2013b), have the same conjoined three-/four-stranded coiled-coil topologies. The significance of this is currently not understood, although some speculation can be made based on comparison with the DLC1/talin R8 complex, which has the same helix arrangement as a talin rod five-helix bundle (Figure 2). The core of the fold is a typical four-helix bundle that is likely to remain stable when the N-terminal α0 helix is removed: the talin R8 four-helix bundle is perfectly stable in the absence of DLC1, and removal of the N-terminal α0 helix from the R10 domain generates a stable four-helix bundle (Gingras et al., 2006; Goult et al., 2010) that is similar to R8. This suggests that under some conditions, talin five-helix domains may exist as four-helix bundles, raising the exciting possibility that removal of the α0 helix might expose cryptic binding sites that can interact with helical regions homologous to the α0 sequence. The VBSs in the talin rod are buried in the hydrophobic core of the helical bundles in which they are contained (Calderwood et al., 2013), and force exerted on talin is required to expose these sites (del Rio et al., 2009; Yao et al., 2014). It is therefore tempting to speculate that force may also play a role in displacing the α0 helix in talin rod five-helix bundles, exposing cryptic binding sites for proteins such as those containing LD motifs.

Although talin is widely recognized as a key player in adhesion complex assembly, the extent of the talin interaction network is unclear, and no comprehensive proteomic study on talin binding partners has been reported. Rather, the majority of studies have concentrated on individual interactions that are often prominent under specific conditions. Experiments in live cells demonstrate that adhesion complex assembly has a high tolerance for deletion of individual proteins, as well as deletions or mutations of individual binding sites. This implies a high level of redundancy in the system, some of which may be due to the multi-site interactions between FA proteins.

EXPERIMENTAL PROCEDURES

Peptides and Protein Preparation

Recombinant wild-type mouse talin1 fragment R7R8 (residues 1,357–1,653) was previously cloned into pET151/D-TOPO expression vector (Gingras et al., 2010). Site-directed R7R8 mutants were produced by overlap extension PCR and subsequent ligation-independent cloning into pOPINB vector (OPPF-UK). Protein was produced in BL21 STAR (DE3) cultured in Luria-Bertani or 2× M9 minimal medium containing 1 g/l ^{15}N-labeled NH_4Cl, and purified using nickel-affinity chromatography followed by ion exchange.

X-Ray Crystallography

Sitting-drop sparse matrix crystallization screens were set up using a 300-μM solution of talin R7R8 fragment in the presence of 8-fold molar excess of DLC1(467–489) peptide. Crystals were obtained in 15% ethanol and 0.1 M Tris (pH 7.4) at 4°C and vitrified in sodium malonate (pH 7) prior to data collection. The DLC1/R7R8 complex was solved using molecular replacement using the structure of the free R7 domain as a template (PDB: 2X0C) (Gingras et al., 2010). Initial electron density maps showed that the position of the R8 domain had changed, and once repositioned and the R7R8 domain modeled, electron density for the DLC1 peptide was clearly visible, as demonstrated in the simulated annealing composite omit map (Figure S1A). Refinement was performed using isotropic B factors, and at the final stage of refinement employed the use of TLS parameters. Data reduction and refinement statistics are shown in Table 1.

NMR Spectroscopy

NMR spectra were collected on Bruker Avance III 600- and 800-MHz spectrometers equipped with CryoProbes. Experiments were performed at 298 K in 20 mM sodium phosphate (pH 6.5) and 50 mM NaCl with 5% (v/v) ^2H_2O. Dissociation constants were evaluated from the ^{1}H,^{15}N-HSQC chemical-shift changes in the titration experiments conducted using 0.1 mM [^{15}N]talin R8 domain. Peptides were added from 5- to 10-mM stock solutions to generate titration points at peptide/protein ratios 0.1, 0.2, 0.5, 0.75, 1, 2, 4, and 8.

Cell-Based Assays

The plasmids expressing GFP-DLC1 and GST fusion proteins with talin rod fragments encoding talin amino acids 1,288–1,646 and 1,453–1,580 (R8) were described previously (Li et al., 2011). The plasmid encoding 1,352–1,580 (R7R8) was engineered by PCR and subcloned into a eukaryotic expression vector, PEBG. HEK293T cells were transfected by Lipofectamine 2000, and DLC1-null lung adenocarcinoma cell lines A549 and H358 cells were transfected by Lipofectamine 3000 according to the manufacturer's instructions (Invitrogen). Cells were co-transfected with plasmids expressing GFP-DLC1 or Paxillin-DDK and GST, GST-talin fragments, or vector at a ratio of 1:2.5. Cells were incubated at 37°C in a humidified 5% CO_2 atmosphere. In vivo pull-down assay, co-immunoprecipitation, immunoblotting, G418 colony growth, soft agar growth, and cell migration assays were described previously (Qian et al., 2009). All experiments were conducted in triplicate.

Ratio Imaging

Talin1 and talin2 knockout cells were generated and cultured as described in Atherton et al. (2015). Transient transfections were performed using Lipofectamine and Plus reagents (Life Technologies) as per the manufacturer's instructions. Cells transfected with GFP-talin proteins were incubated overnight on glass-bottomed dishes (MatTek), fixed with 4% paraformaldehyde and permeabilized with 0.5% Triton X-100 (Sigma). Samples were incubated with the primary antibody for 60 min and then washed thrice with PBS. Secondary antibody staining followed the same procedure. Fixed samples were imaged using a Delta Vision RT microscope (Applied Precision) equipped with a 60×/1.42 Plan Apo oil-immersion objective (Zeiss). Images were acquired with a CoolSnap HQ camera (Photometrics). Images were background subtracted, a region of interest was selected around an individual peripheral adhesion (five per cell), and the integrated density measured for both channels. Dividing the values from paxillin or DLC1 by talin then produced a ratio.

Further details can be found in Supplemental Experimental Procedures.

ACCESSION NUMBERS

The coordinates for the structure of the talin R8/DLC1 complex have been deposited in the PDB under accession code PDB: 5FZT. Backbone chemical shifts of the talin R8 have been deposited in the Biological Magnetic Resonance Bank under accession code BMRB: 19339.

SUPPLEMENTAL INFORMATION

Supplemental Information includes Supplemental Experimental Procedures and six figures and can be found with this article online at http://dx.doi.org/10.1016/j.str.2016.04.016.

AUTHOR CONTRIBUTIONS

T.Z., X.Q., B.T.G., D.J., and I.L.B. conducted the experiments with contributions from T.B.A., T.Z., X.Q., B.T.G., D.J., C.B., D.R.L., and I.L.B. analyzed the data. C.B., D.R.C., D.R.L., and I.L.B. designed the research. T.Z., D.R.C., D.R.L., and I.L.B. wrote the paper with contributions from X.Q., B.T.G., and C.B. I.L.B. supervised and directed the project.

ACKNOWLEDGMENTS

T.Z. was supported by the Biotechnology and Biological Sciences Research Council (BBSRC) DTP fellowship. B.T.G is funded by BBSRC (BB/N007336/1). The C.B. laboratory is part of the Wellcome Trust Centre for Cell-Matrix Research, University of Manchester, which is supported by core funding from the Wellcome Trust (grant number 088785/Z/09/Z). D.J. is supported by a BBSRC DTP studentship. We thank the staff of the Bioimaging facility at the University of Manchester for help with imaging. The X-ray data were collected at the Diamond Light Source under Liverpool BAG allocation.

Received: November 4, 2015
Revised: April 4, 2016
Accepted: April 5, 2016
Published: June 2, 2016

REFERENCES

Alam, T., Alazmi, M., Gao, X., and Arold, S.T. (2014). How to find a leucine in a haystack? Structure, ligand recognition and regulation of leucine-aspartic acid (LD) motifs. Biochem. J. *460*, 317–329.

Atherton, P., Stutchbury, B., Wang, D.Y., Jethwa, D., Tsang, R., Meiler-Rodriguez, E., Wang, P., Bate, N., Zent, R., Barsukov, I.L., et al. (2015). Vinculin controls talin engagement with the actomyosin machinery. Nat. Commun. *6*, 10038.

Calderwood, D.A., Campbell, I.D., and Critchley, D.R. (2013). Talins and kindlins: partners in integrin-mediated adhesion. Nat. Rev. Mol. Cell Biol. *14*, 503–517.

Carisey, A., Tsang, R., Greiner, A.M., Nijenhuis, N., Heath, N., Nazgiewicz, A., Kemkemer, R., Derby, B., Spatz, J., and Ballestrem, C. (2013). Vinculin regulates the recruitment and release of core focal adhesion proteins in a force-dependent manner. Curr. Biol. *23*, 271–281.

Chang, Y.C., Zhang, H., Franco-Barraza, J., Brennan, M.L., Patel, T., Cukierman, E., and Wu, J.H. (2014). Structural and mechanistic insights into the recruitment of talin by RIAM in integrin signaling. Structure *22*, 1810–1820.

del Rio, A., Perez-Jimenez, R., Liu, R., Roca-Cusachs, P., Fernandez, J.M., and Sheetz, M.P. (2009). Stretching single talin rod molecules activates vinculin binding. Science *323*, 638–641.

Desiniotis, A., and Kyprianou, N. (2011). Significance of talin in cancer progression and metastasis. Int. Rev. Cell Mol. Biol. *289*, 117–147.

Devreotes, P., and Horwitz, A.R. (2015). Signaling networks that regulate cell migration. Cold Spring Harb. Perspect. Biol. *7*, a005959.

Fillingham, I., Gingras, A.R., Papagrigoriou, E., Patel, B., Emsley, J., Critchley, D.R., Roberts, G.C.K., and Barsukov, I.L. (2005). A vinculin binding domain

from the talin rod unfolds to form a complex with the vinculin head. Structure *13*, 65–74.

Gardel, M.L., Schneider, I.C., Aratyn-Schaus, Y., and Waterman, C.M. (2010). Mechanical integration of actin and adhesion dynamics in cell migration. Annu. Rev. Cell Dev. Biol. *26*, 315–333.

Gingras, A.R., Vogel, K.P., Steinhoff, H.J., Ziegler, W.H., Patel, B., Emsley, J., Critchley, D.R., Roberts, G.C.K., and Barsukov, I.L. (2006). Structural and dynamic characterization of a vinculin binding site in the talin rod. Biochemistry *45*, 1805–1817.

Gingras, A.R., Bate, N., Goult, B.T., Hazelwood, L., Canestrelli, I., Grossmann, J.G., Liu, H., Putz, N.S., Roberts, G.C., Volkmann, N., et al. (2008). The structure of the C-terminal actin-binding domain of talin. EMBO J. *27*, 458–469.

Gingras, A.R., Bate, N., Goult, B.T., Patel, B., Kopp, P.M., Emsley, J., Barsukov, I.L., Roberts, G.C., and Critchley, D.R. (2010). Central region of talin has a unique fold that binds vinculin and actin. J. Biol. Chem. *285*, 29577–29587.

Goult, B.T., Gingras, A.R., Bate, N., Barsukov, I.L., Critchley, D.R., and Roberts, G.C. (2010). The domain structure of talin: residues 1815-1973 form a five-helix bundle containing a cryptic vinculin-binding site. FEBS Lett. *584*, 2237–2241.

Goult, B.T., Xu, X.P., Gingras, A.R., Swift, M., Patel, B., Bate, N., Kopp, P.M., Barsukov, I.L., Critchley, D.R., Volkmann, N., et al. (2013a). Structural studies on full-length talin1 reveal a compact auto-inhibited dimer: implications for talin activation. J. Struct. Biol. *184*, 21–32.

Goult, B.T., Zacharchenko, T., Bate, N., Tsang, R., Hey, F., Gingras, A.R., Elliott, P.R., Roberts, G.C., Ballestrem, C., Critchley, D.R., et al. (2013b). RIAM and vinculin binding to talin are mutually exclusive and regulate adhesion assembly and turnover. J. Biol. Chem. *288*, 8238–8249.

Hayashi, I., Vuori, K., and Liddington, R.C. (2002). The focal adhesion targeting (FAT) region of focal adhesion kinase is a four-helix bundle that binds paxillin. Nat. Struct. Biol. *9*, 101–106.

Hoellerer, M.K., Noble, M.E.M., Labesse, G., Campbell, I.D., Werner, J.M., and Arold, S.T. (2003). Molecular recognition of paxillin LD motifs by the focal adhesion targeting domain. Structure *11*, 1207–1217.

Lawson, C., and Schlaepfer, D.D. (2012). Integrin adhesions: Who's on first? What's on second? Connections between FAK and talin. Cell Adh. Migr. *6*, 302–306.

Lawson, C.D., and Burridge, K. (2014). The on-off relationship of Rho and Rac during integrin-mediated adhesion and cell migration. Small GTPases *5*, e27958.

Lawson, C., Lim, S.T., Uryu, S., Chen, X.L., Calderwood, D.A., and Schlaepfer, D.D. (2012). FAK promotes recruitment of talin to nascent adhesions to control cell motility. J. Cell Biol. *196*, 223–232.

Lee, H.S., Lim, C.J., Puzon-McLaughlin, W., Shattil, S.J., and Ginsberg, M.H. (2009). RIAM activates integrins by linking talin to ras GTPase membrane-targeting sequences. J. Biol. Chem. *284*, 5119–5127.

Li, G., Du, X., Vass, W.C., Papageorge, A.G., Lowy, D.R., and Qian, X. (2011). Full activity of the deleted in liver cancer 1 (DLC1) tumor suppressor depends on an LD-like motif that binds talin and focal adhesion kinase (FAK). Proc. Natl. Acad. Sci. USA *108*, 17129–17134.

Lupas, A.N., and Gruber, M. (2005). The structure of alpha-helical coiled coils. Adv. Protein Chem. *70*, 37–78.

Moutevelis, E., and Woolfson, D.N. (2009). A periodic table of coiled-coil protein structures. J. Mol. Biol. *385*, 726–732.

Papagrigoriou, E., Gingras, A.R., Barsukov, I.L., Bate, N., Fillingham, I.J., Patel, B., Frank, R., Ziegler, W.H., Roberts, G.C.K., Critchley, D.R., et al. (2004). Activation of a vinculin-binding site in the talin rod involves rearrangement of a five-helix bundle. EMBO J. *23*, 2942–2951.

Qian, X., Li, G., Vass, W.C., Papageorge, A., Walker, R.C., Asnaghi, L., Steinbach, P.J., Tosato, G., Hunter, K., and Lowy, D.R. (2009). The Tensin-3 protein, including its SH2 domain, is phosphorylated by Src and contributes to tumorigenesis and metastasis. Cancer Cell *16*, 246–258.

Roca-Cusachs, P., Iskratsch, T., and Sheetz, M.P. (2012). Finding the weakest link: exploring integrin-mediated mechanical molecular pathways. J. Cell Sci. *125*, 3025–3038.

Seguin, L., Desgrosellier, J.S., Weis, S.M., and Cheresh, D.A. (2015). Integrins and cancer: regulators of cancer stemness, metastasis, and drug resistance. Trends Cell Biol. *25*, 234–240.

Wang, S.J., Watanabe, T., Matsuzawa, K., Katsumi, A., Kakeno, M., Matsui, T., Ye, F., Sato, K., Murase, K., Sugiyama, I., et al. (2012). Tiam1 interaction with the PAR complex promotes talin-mediated Rac1 activation during polarized cell migration. J. Cell Biol. *199*, 331–345.

Wehrle-Haller, B. (2012). Assembly and disassembly of cell matrix adhesions. Curr. Opin. Cell Biol. *24*, 569–581.

Yang, J., Zhu, L., Zhang, H., Hirbawi, J., Fukuda, K., Dwivedi, P., Liu, J., Byzova, T., Plow, E.F., Wu, J., et al. (2014). Conformational activation of talin by RIAM triggers integrin-mediated cell adhesion. Nat. Commun. *5*, 5880.

Yao, M., Goult, B.T., Chen, H., Cong, P., Sheetz, M.P., and Yan, J. (2014). Mechanical activation of vinculin binding to talin locks talin in an unfolded conformation. Sci. Rep. *4*, 4610.

Zhang, X., Jiang, G., Cai, Y., Monkley, S.J., Critchley, D.R., and Sheetz, M.P. (2008). Talin depletion reveals independence of initial cell spreading from integrin activation and traction. Nat. Cell Biol. *10*, 1062–1068.

Structure, Volume *24*

Supplemental Information

LD Motif Recognition by Talin:

Structure of the Talin-DLC1 Complex

Thomas Zacharchenko, Xiaolan Qian, Benjamin T. Goult, Devina Jethwa, Teresa B. Almeida, Christoph Ballestrem, David R. Critchley, Douglas R. Lowy, and Igor L. Barsukov

Supplementary information

LD-motif recognition by talin: structure of the talin-DLC1 complex

Thomas Zacharchenko, Xiaolan Qian, Benjamin T. Goult, Devina Jethwa, Teresa B. Almeida, Christoph Ballestrem, David R. Critchley, Douglas R. Lowy and Igor L. Barsukov

Supplementary Figures

Supplementary Methods

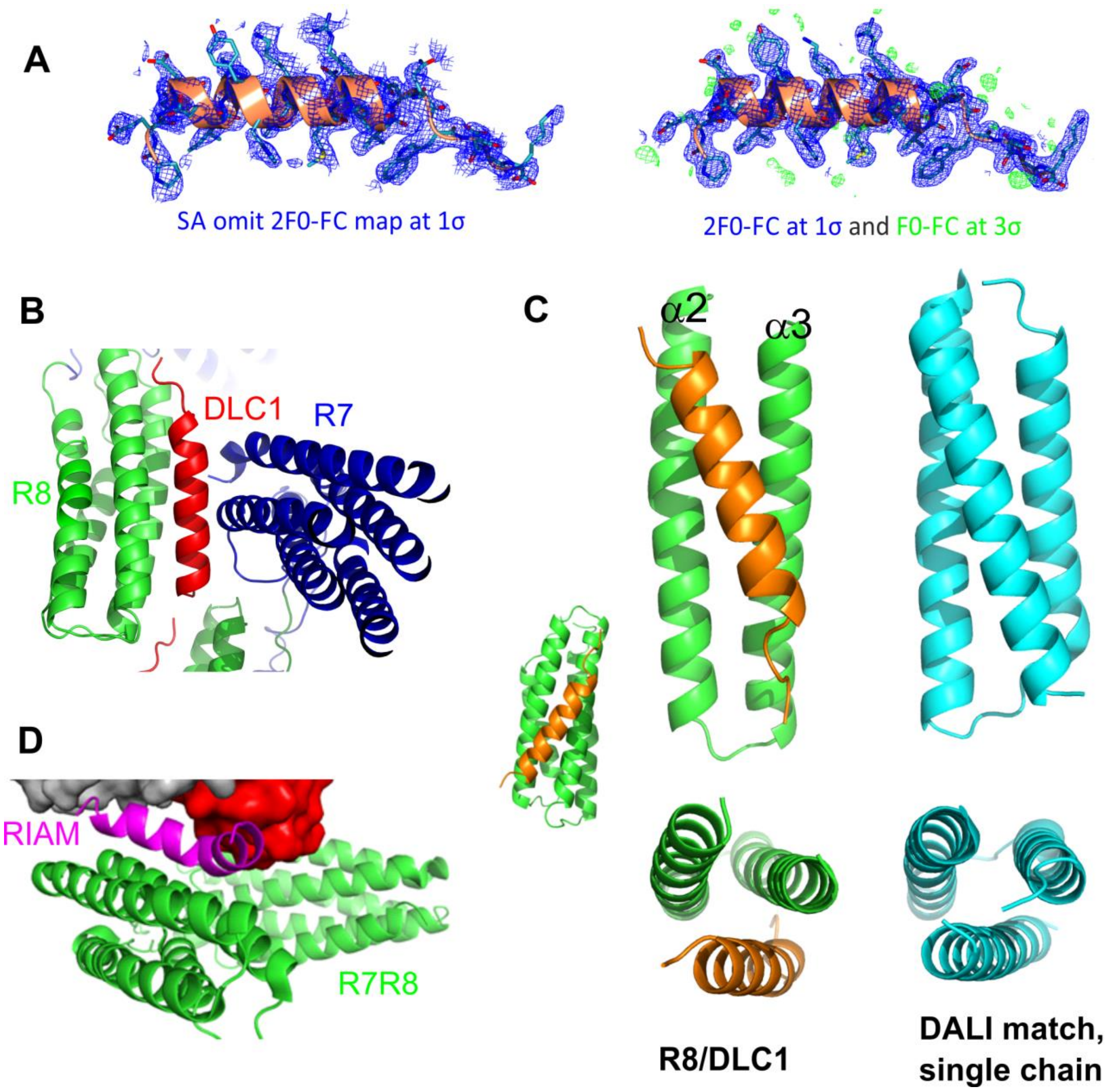

Figure S1, related to Figures 2 and 3. Structural characteristics of R7R8/DLC1 complex

(A) Structure of DLC1 peptide is well defined in the complex. Simulated annealing composite omit map 2F0-FC shown at 1 σ (blue, left) and the refined 2F0-FC map (blue, right) of the DLC1 LD-motif shown at 1 σ superimposed on the structure of the DLC1 peptide in the complex (orange) show a good match between the structure and the electron density.

(B) Crystal packing contacts in the R7R8/DLC1 complex. Outer surface of the DCL1 helix (red) makes limited contacts with the edge of the R7 domain of the symmetry-related molecule.

(C) Triple-helix structure in the talin R8/DLC1 complex. Similarity between the arrangement of the α2-α3 hairpin of the talin R8 and DLC1 fragment from the R8/DLC1 complex (left) and the single-chain, triple-helix structure of BAG family molecular chaperone regulator 4 (right; PDB: 4HWH), identified as a close match by DALI server (http://ekhidna.biocenter.helsinki.fi/dali_server/)

(D) Crystal packing contacts in the talin R7R8/RIAM complex (PDB: 4W8P). RIAM helix (magenta) bound to the R7R8 talin fragments (green) makes contacts with the symmetry-related talin R7 domain (red) that may induce the kink in the RIAM helix. A straight helix would clash with the symmetry-related R7.

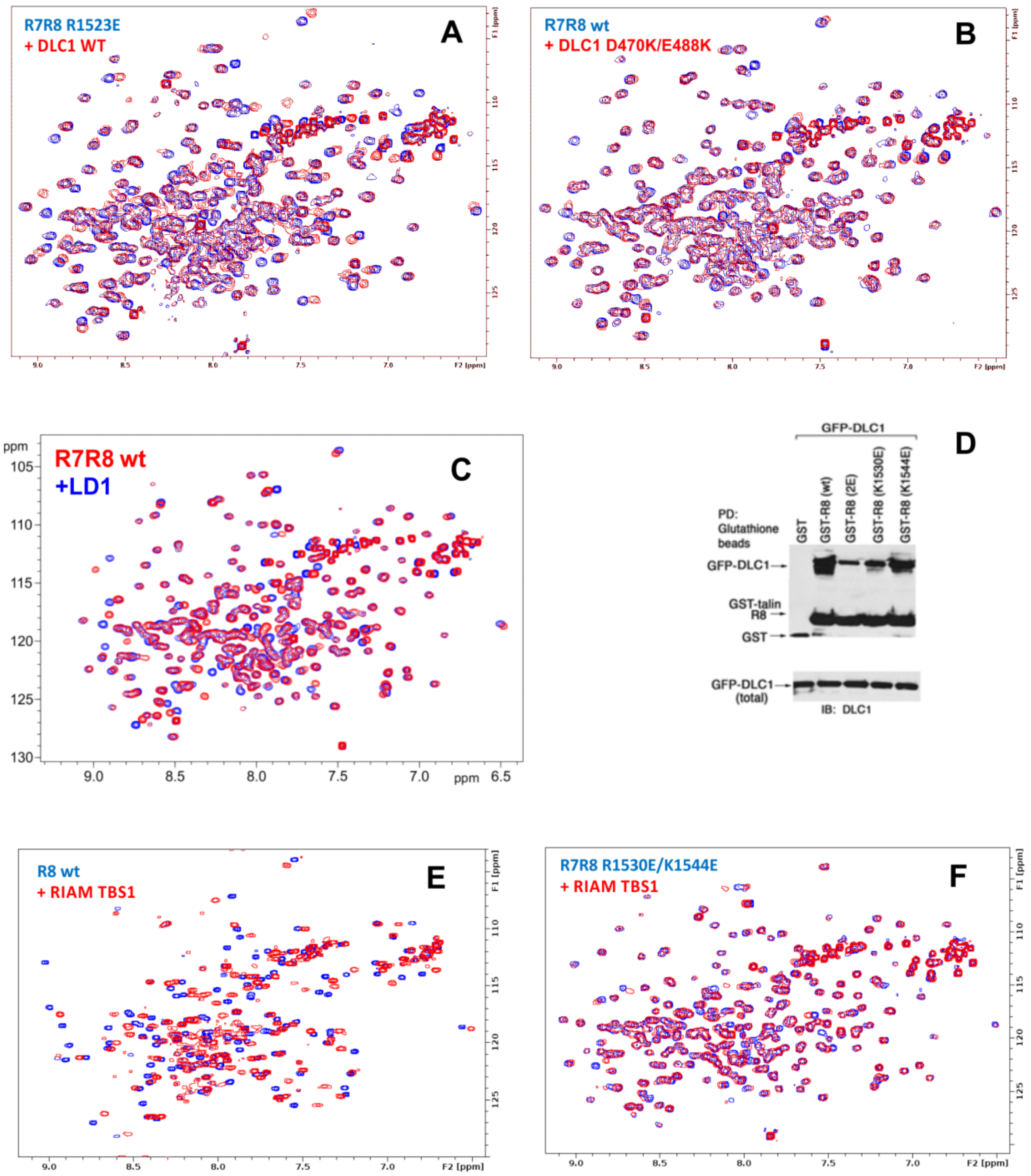

Figure S2, related to Figures 4 and 5. Point mutations disrupt the interaction between talin R8 and DLC1 or paxillin.

(A) Superposition of the HSQC spectra of 0.2 mM K1523E mutant of talin R7R8 free (blue) and in the presence of 0.8 mM DLC1(461-489) (red).

(B) Superposition of the HSQC spectra of 0.2 mM talin R7R8 free (blue) and in the presence of 0.8 mM D470K/E488K DLC1(461-489) mutant (red).

(C) Superposition of the HSQC spectra of 0.2 mM K1544E mutant of talin R7R8 free (red) and in the presence of 0.8 mM of paxillin LD1 (blue).

(D) GST-talin R1530E binds DLC1 less well than the K1544E mutant. Wild-type or mutant GST-R8 talin fragment were co-transfected with GFP-DLC1 into 293T cells. Cell extracts were subjected

to pull-down assay with glutathione beads followed by immunoblotting with anti-GST and anti-DLC1 on the same membrane (top). The transfected GFP-DLC1 in each sample is shown by the anti-DLC1 blot (bottom) as a loading control.

(E) Superposition of the HSQC spectra of 0.2 mM of talin R7R8 free (blue), and in the presence of 0.2 mM of RIAM TBS1 (red).

(F) Superposition of the HSQC spectra of 0.2 mM of R1530E/K1544E mutant of talin R7R8 free (blue), and in the presence of 0.2 mM of RIAM TBS1 (red).

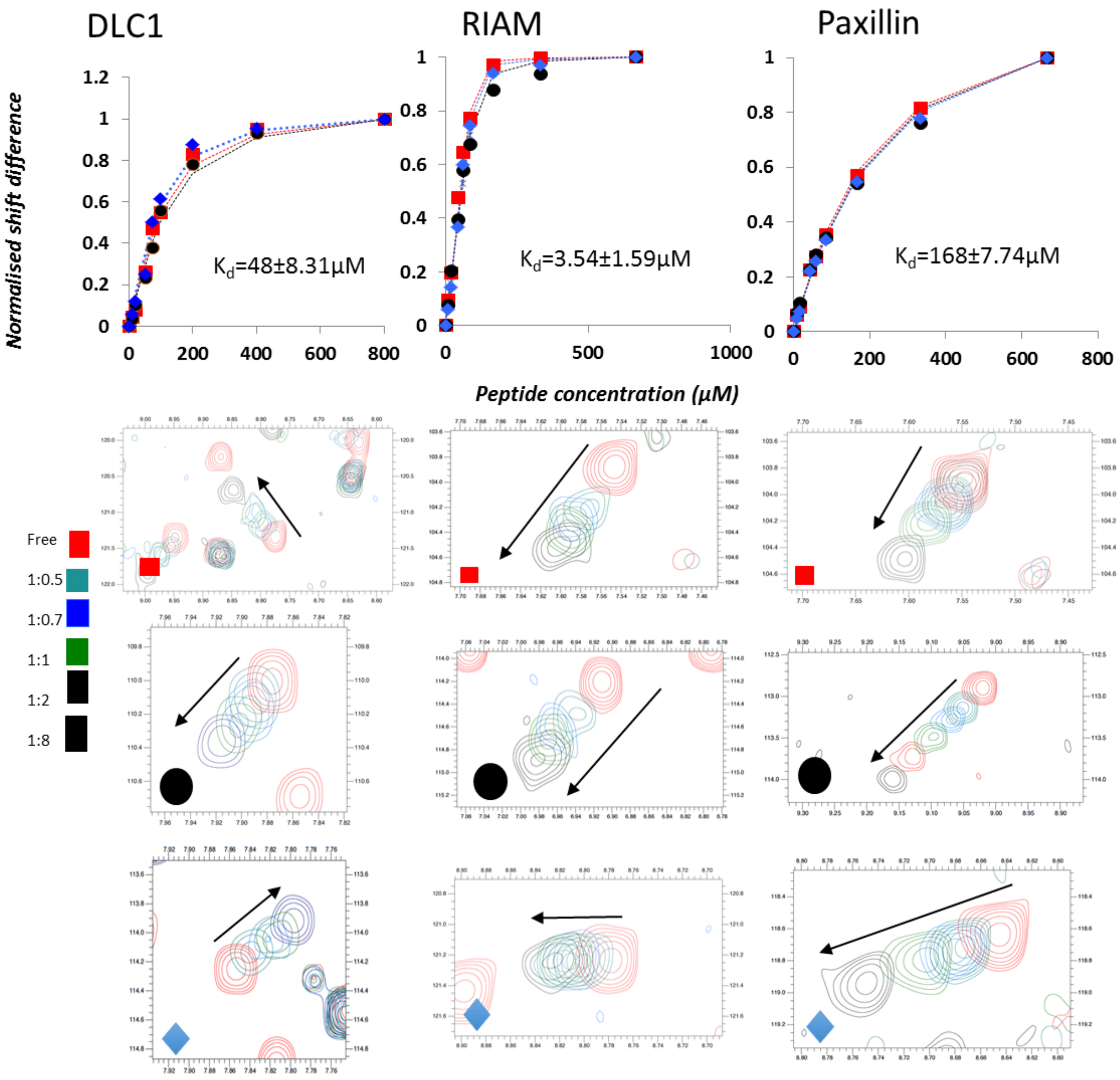

Figure S3, related to Figures 1 and 5. Dissociation constants for DLC1, RIAM and paxillin interactions with talin R8.

(A) Examples of the chemical shifts changes in the $^{1}H,^{15}N$-HSQC spectra of talin R8 on the addition of the peptide ligands used to determine K_d values. Symbols represent experimental chemical shift differences. Solid lines of the corresponding colours show the fitted curves. For each curve, chemical shift changes are normalised to the last value.

(B) Superposition of the HSQC spectra for each of the cross-peaks used in the fitting in (A). Limited resonance broadening throughout the titration confirms fast exchange regime for the cross-peaks used in the K_d measurements.

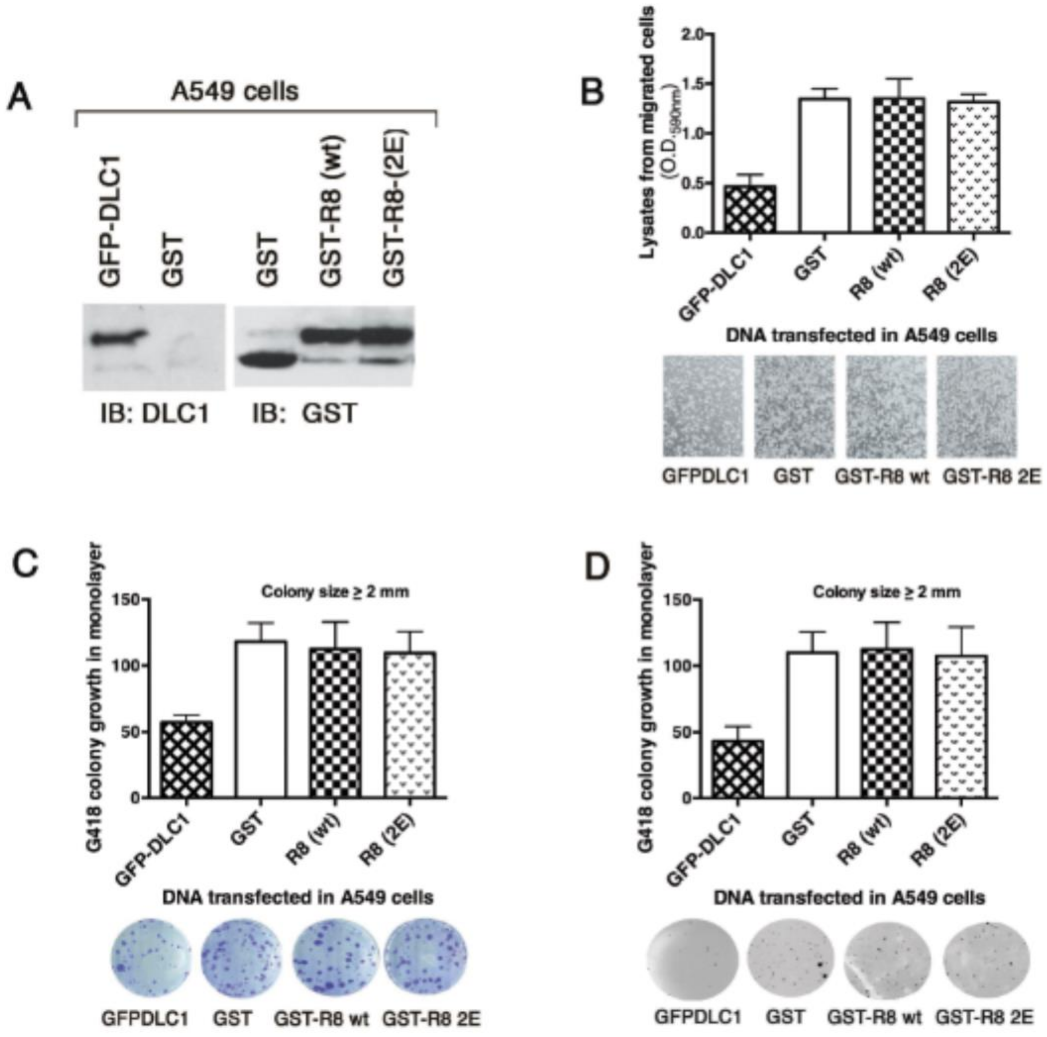

Figure S4, related to Figure 6. Expression of GST-talin R8 alone is insufficient to cause significant biological changes in A549 cells.

(A) Co-transfection of A549 cells with GFP-DLC1 and either GST, GST-R8 wt, or GST-R8 2E constructs. Six days after transfection, A549 cell extracts were analysed by anti-DLC1 (left) and anti-GST (right) immunoblotting (IB). Note – GST constructs were purified on glutathione beads prior to immunoblotting, and were expressed at similar levels.

(B) Cell migration. Transfected cells were analysed using a transwell migration assay as described in methods. The lysates from migrated cells were quantitated (top), and representative microscopic images of the migrated cells are shown (bottom). As expected, GFP-DLC1 suppressed cell migration whereas the the biological activity of the talin R8 wt and 2E mutant was similar to that of the GST negative control.

(C) G418 colony growth. The transfected cells were cultured in G418 for 3 weeks, and the colonies were counted and quantitated (top). Representative stained colonies are shown (bottom). As expected, GFP-DLC1 suppressed colony formation whereas the biological activity of the talin R8 wt and 2E mutant was similar to that of the GST negative control.

(D) Growth in soft agar. The transfected cells were grown 3 weeks in soft agar, and the colonies were counted and quantitated (top). Representative stained whole dishes are shown (bottom). As expected, GFP-DLC1 suppressed growth in soft agar whereas the biological activity of the talin R8 wt and 2E mutant was similar to that of the GST negative control.

The results in (B)-(D) are represented as means over three experiments ± SD.

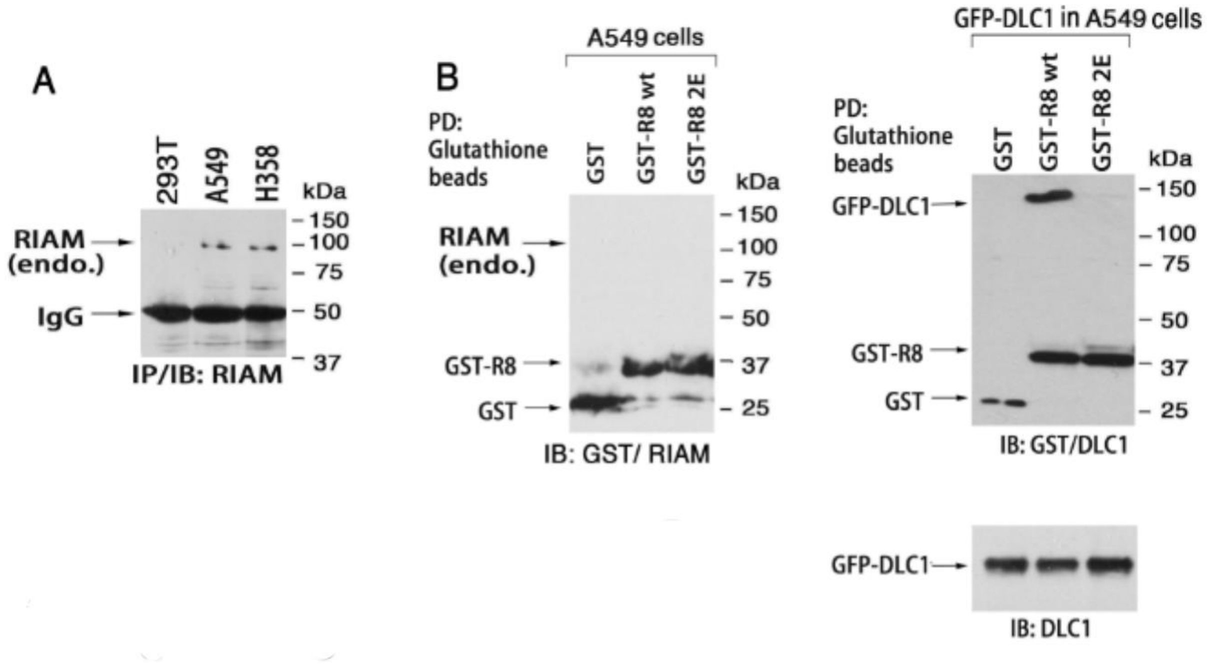

Figure S5, related to figure 6. RIAM is expressed at low levels in A549 cells and does not form a detectable complex with GST-talin R8.

(A) The expression of endogenous RIAM in cell lines. Cell extracts were subjected to immunoprecipitation followed by immunoblotting with anti-RIAM antibody.

(B) A complex between GST-talin R8 and endogenous RIAM is undetectable in A549 cells. A549 cells were co-transfected with GFP-DLC1 and the GST constructs indicated. Cell extracts were subject to a glutathione bead pull down assay followed by immunoblotting with anti-GST and anti-RIAM antibodies on the same membrane (left). No complex between GST-talin R8 and RIAM could be detected. Under the same conditions, wild-type GST-R8 pulled-down the co-transfected GFP-DLC1; the immunoblot with anti-GST and anti-DLC1 on the same membrane is shown (right). The amount of transfected GFP-DLC1 in each sample (loading control) is shown by the anti-DLC1 blot (bottom).

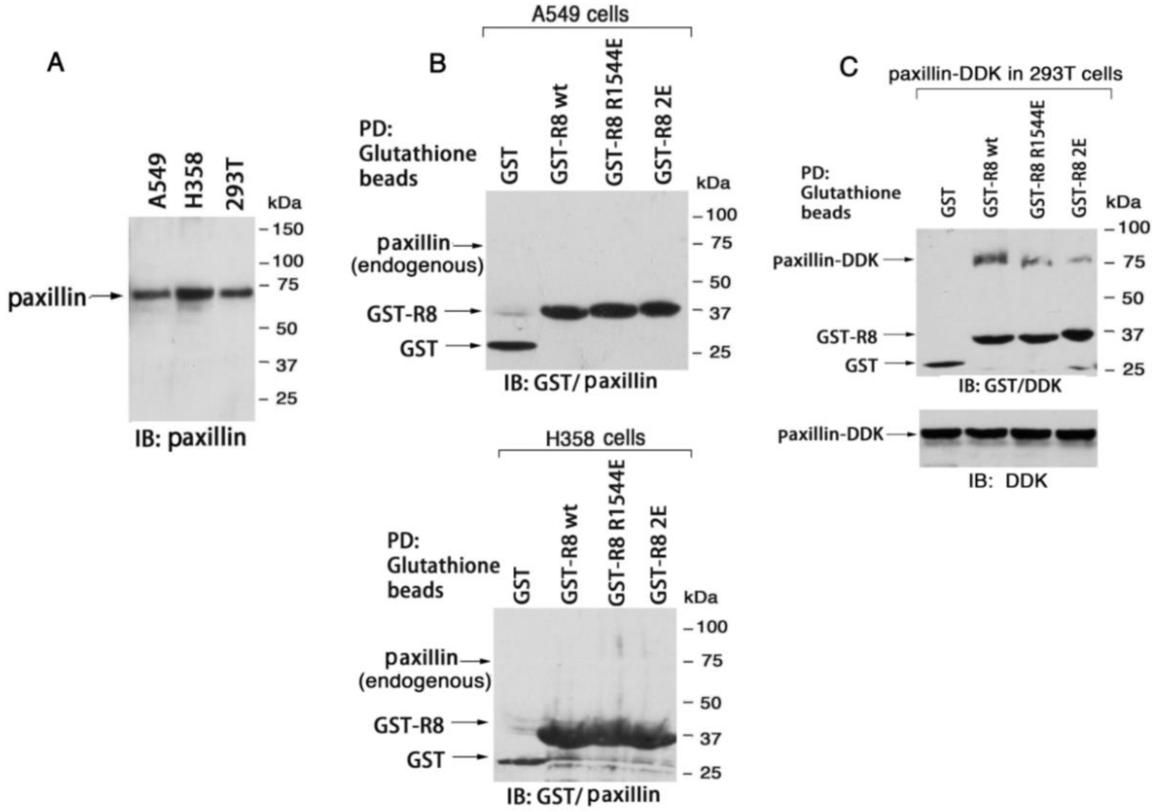

Figure S6, related to figure 5. Binding of GST-talin R8 to endogenous paxillin is undetectable in A549 and H358 cells.

(A) The expression of endogenous paxillin in cell lines. Cell extracts were assayed by immunoblotting with anti-paxillin antibody.

(B) A complex of GST-talin R8 with endogenous paxillin is undetectable in A549 (top) and H358 (bottom) cells. Cells were transfected with the GST constructs indicated, and cell extracts subjected to a glutathione bead pull down assay followed by immunoblotting with anti-GST and anti-paxillin antibodies on the same membrane.

(C) Wild-type GST-talin R8 forms a complex with transfected paxillin in 293T cells. Extracts of 293 cells co-transfected with the GST constructs indicated and paxillin-DDK (OriGene) were subjected to pull-down assays with glutathione beads followed by immunoblotting with anti-GST and anti-DDK antibodies on the same membrane. While GST-talin R8 wt formed a complex with paxillin-DDK, the amount of paxillin complexed to the GST-talin R8 mutants was significantly reduced. The transfected paxillin-DDK in each sample is shown by the anti-DDK blot (bottom) as a loading control.

Supplementary Methods

Peptides and protein preparation – Recombinant wild-type mouse talin1 fragment R7R8 (residues 1357-1653) was previously cloned into pET151/D-TOPO expression vector (Invitrogen), encoding an N-terminal hexa-histidine tag (Gingras et al., 2010). Site directed R7R8 mutants were produced by overlap extension PCR, and subsequent ligation-independent cloning into pOPINB vector (OPPF-UK); constructs were verified by sequencing. Both vectors include N-terminal hexa-histidine tag followed by TEV protease cleavage site.

Proteins were expressed and purified as described previously (Gingras et al., 2010). Briefly, protein was produced in BL21 STAR (DE3) cultured in LB or 2xM9 minimal medium containing 1 g/L of ^{15}N-labelled NH_4Cl. Cells were grown at 37 ^{o}C to an OD_{600} of 0.6, cooled to 18 ^{o}C and induced using 0.5 mM IPTG for 16 hours. Recombinant His-tagged protein was purified by nickel-affinity chromatography following standard protocol. The tag was removed by cleavage with TEV protease, followed by the reverse purification. Protein was further purified using anion exchange chromatography using a 5 ml Hi-Trap QFF column (GE Healthcare).

Paxillin LD1 (^{1}MDDLDALLADLESTTS^{16}) and LD2 (^{141}NLSELDRLLLELNAVQHNPP^{160}) (Mus musculus) were synthesised at the proteomics facility at University of Nottingham; DLC1 TBS peptides (^{467}PELDDILYHVKGMQRIVNQWSEK^{489} and ^{461}ENEDIFPELDDILYHVKGMQRIVNQWSEK^{489}) (Homo sapiens) were synthesised by GL Biochem (Shanghai, China). Peptides were purified to >95% by reverse-phase chromatography and sequence analysed by mass spectrometry.

X-ray crystallography – Diffraction data were collected at beamline i03 (Diamond light source) at a wavelength of 0.97Å. Intensities were integrated and scaled using iMOSFILM and SCALA (Evans, 2006), with 5% of reflections randomly isolated from refinement. The DLC1-R7R8 complex was solved using molecular replacement using the structure of the free R7 domain as a template (PDB: 2X0C) (Gingras et al., 2010). Molecular replacement was performed using PHASER (McCoy et al., 2007) and modelling was performed using COOT 0.8.1 (Emsley and Cowtan, 2004).

Initial electron density maps showed that the position of the R8 domain had changed, and once repositioned, and the R7R8 domain modelled, electron density for the DLC1 peptide was clearly visible, as demonstrated in the simulated annealing composite omit map (Supplementary Figure S1A). Electron density was visible for the entire helix and allowed unambiguous assignment of electron density. Refinement was performed using isotropic B-factors, and at the final stage of refinement employed the use of TLS parameters determined by the TLS motion determination server (Painter and Merritt, 2006), TLS groups included residues 1354-1466 and 1467-1659 of

R7R8 and the entire DLC1 peptide, 467-469. Refinement and validation were performed using PHENIX 1.10 (Adams et al., 2010). Data reduction and refinement statistics are shown in Table 1.

NMR Spectroscopy - Spectra were processed with TopSpin (Bruker) and analysed using CCPN Analysis (Vranken et al., 2005). Resonance assignment was carried out as described previously (Goult et al., 2008). The changes in chemical shift of ^{15}N-talin R8 domain were calculated from ^{1}H,^{15}N-HSQC spectra as $\Delta\delta = \sqrt{(\Delta\delta H^2 + (\Delta\delta N * 0.15)^2)}$. The cross-peaks corresponding to the bound state were assigned by following chemical shift changes throughout the R8 titration with the increasing amount of peptides.

Dissociation constants were evaluated from the ^{1}H,^{15}N-HSQC chemical shift changes in the titration experiments conducted using 0.1 mM ^{15}N-talin R8 domain. Peptides were added from 5-10 mM stock solutions to generate titration points at 0.1, 0.2, 0.5, 0.75, 1, 2, 4 and 8 peptide:protein ratios. Concentrations of the DLC1 and RIAM peptides were determined from UV absorbance at 280 nm. Concentrations of the paxillin peptide that lacks UV-active aromatic residues was determined by NMR from the comparison of the integral intensities of the well-separated signals of the methyl groups of the paxillin and RIAM peptides following the Eretic2 procedure of TopSpin (Bruker). Concentration of the R8 domain that lacks UV-active aromatic residues was estimated from the comparison with the spectra of the R3 domain that has similar molecular weight and shape using the calibration procedure described in (Wider and Dreier, 2006). Dissociation constants were determined by fitting the chemical shift changes to the equation:

$$\Delta(H,N) = \Delta(H,N)_0 \frac{[P] + [L] + Kd - \sqrt{([P] + [L] + Kd)^2 - 4[P][L]}}{2[P]}$$

where $\Delta(H,N)_0$ is the weighted chemical shift difference at saturation, [P] and [L] are protein and ligand concentrations, respectively. Data were fitted independently for the well-resolved peaks and the average K_d was calculated from 3 different peaks with the lowest standard deviations of the fits. For DLC1 and RIAM only signals in fast exchange regime were selected for the analysis.

DNA Constructs and transfection - The plasmids expressing GFP-DLC1 and GST fusion proteins with talin rod fragments encoding talin amino acids 1288-1646 and 1453-1580 (R8) were described previously (Li et al., 2011). The plasmid encoding 1352-1580 (R7R8) was engineered by PCR, and subcloned into a eukaryotic expression vector, PEBG (Anborgh et al., 1999), using BamHI and NotI sites. The point mutations, K1530E/K1544E, in R8 were made individually and in combination by site-directed mutagenesis using a mutagenesis kit (Agilent). Human Paxillin-DDK (PXN) plasmid was from OriGene.

HEK 293T cells were transfected by lipofectamine 2000 and DLC1-null lung adenocarcinoma cell lines A549 and H358 cells were transfected by lipofectamine 3000 according to manufacturer instructions (Invitrogen). Cells were co-transfected with plasmids expressing GFP-DLC1 or Paxillin-DDK and GST, GST-talin fragments, or vector at a ratio of 1:2.5. Cells were incubated at 37 ^{o}C in a humidified 5% CO_2 atmosphere.

In vivo pull-down assay, co-immunoprecipitation, and immunoblotting - As described previously (Qian et al., 2009), two days after transfection, cell extracts were collected using golden lysis buffer (GLB: 20 mM Tris pH 7.9, 137 mM NaCl, 10% glycerol, 1% Triton, 5 mM EDTA, 1 mM EGTA, 1 mM Na_3VO_4, 10 mM NaF, 1 mM sodium pyrophosphate, 0.5 mM β-glycerophosphate, and protease inhibitor cocktail tablet (Roche)). The cleared supernatants were collected, and the amount of protein estimated by BCA kit (Pierce). Equal amounts of protein from cell extracts were used for pull-down assays by adding 25 µl of glutathione Sepharose-4B slurry (GE Healthcare) and rotating for 3 h at 4 °C. The pellets were washed once with GLB, once with high salt HNTG (20 mM Hepes, 500 mM NaCl, 0.1% Triton-X, 10% glycerol), and twice with low salt HNTG (20 mM Hepes, 150 mM NaCl, 0.1% Triton, 10% glycerol), and incubated with Laemmli sample buffer. After separating the protein samples in SDS-PAGE, the transferred membranes were used for detecting the pull-down proteins by antibody against GST (Santa Cruz), DLC1 (BD Biosciences) and RIAM (Boster Biological Technology Co.) or DDK (OriGene). For co-immunoprecipitation experiments of GFP-DLC1 with endogenous talin, the saved supernatants from each pull-down sample were incubated with anti-talin antibody (Sigma Aldrich). 25 µl of Protein A/G slurry (Pierce) were added to each immune reaction and rotated overnight at 4 °C. The immuno-pellets were washed four times as described for the pull-down assay. Separation of protein samples by SDS-PAGE was followed by immunoblotting using anti-DLC1 or anti-talin antibody. For each blot, horseradish peroxidase-conjugated anti-rabbit or anti-mouse immunoglobulin G (GE Healthcare) was used for the second reaction at 1:10,000 dilution. Immunocomplexes were visualised by enhanced chemiluminescence (ECL), using an ECL kit (GE Healthcare).

G418 colony growth, soft agar growth and cell migration assays - As described previously (Qian et al., 2009), transfected A549 cells were counted, and equal numbers of cells were seeded in triplicate 60 mm dishes (5×10^5 per well) overnight, and cultured with RPMI-1640 media containing 0.9 mg/ml G418 (Invitrogen) for 3 weeks. The G418 resistant colonies were stained by 0.5% crystal violet and counted for graph using Prism. For soft agar colony assays, 1×10^5 cells were mixed with complete medium containing 0.4% agar (Difco) and grown in 60 mm dishes over a thin layer of 0.6% basal agar. Cells were grown for 2-3 weeks, and colonies were photographed microscopically and quantified by a colony counter. Transwell cell migration assays were performed with 6.5 mm

diameter Falcon cell culture inserts (8 µm pore size; Thermo Fisher) precoated with 0.01% gelatin, in 24 well cell culture plates. Cells were trypsinized and resuspended in serum-free media, then transferred to the upper chamber (5×10^4 cells in 350 µl); 800 µl of media containing 10% FBS were added to the lower chamber. After incubation for 24 h, cells remaining on the upper surface of the filter were removed with a cotton swab; cells that had migrated to the lower surface were fixed, stained with 0.5% crystal violet for 10 min, destained, visualised microscopically, and photographed. The migrated cells were then solubilised overnight with 1% Triton-X-100. The collected lysates were quantified colorometrically in a spectrophotometer using OD_{590nm}.

Ratio imaging - Talin1 and talin2 knock out cells were generated and cultured as described in (Atherton et al., 2015). Transient transfections were performed using Lipofectamine and Plus reagents (Life Technologies) as per the manufacturer's instructions.

Mouse anti-paxillin antibody (clone 349/Paxillin) (BD Transduction Laboratories, Europe) and Goat-anti DLC-1 (sc-32931, Santa Cruz Biotechnology) were diluted (1:500) in 1% Bovine Serum Albumin (BSA) (cat: V9131, Sigma, UK). Dylight 594-conjugated AffiniPure Donkey Anti-Mouse IgG (cat: 715-585-150, Jackson ImmunoResearch, USA) and Dylight 594-conjugated AffiniPure Donkey Anti-Goat IgG (cat: 705-586-147, Jackson ImmunoResearch, USA) were used as a secondary antibodies, diluted in 1% BSA (1:500).

Cells transfected with GFP-talin proteins were incubated overnight on glass bottom dishes (MatTek), fixed with 4% paraformaldehyde and permeabilised with 0.5% Triton X-100 (Sigma). Samples were incubated with the primary antibody for 60 min, and then washed thrice with PBS. Secondary antibody staining followed the same procedure. Fixed samples were imaged using a Delta Vision RT microscope (Applied Precision) equipped with a 60 × /1.42 Plan Apo oil immersion objective (Zeiss). Images were acquired with a CoolSnap HQ camera (Photometrics).

Image analysis was carried out using Fiji ImageJ (version 1.48d) software. All cells analysed had low to intermediate levels of talin. Expression levels were determined by fluorescence intensities measured across a large number of cells exposed to the same amount of fluorescent light. For ratio quantification, GFP-talin and DLC1 or paxillin immunofluorescence images were background subtracted, a region of interest was selected around an individual peripheral adhesion (5 per cell) and the integrated density measured for both channels. Dividing the values from paxillin or DLC1 by talin then produced a ratio.

References

Adams, P.D., Afonine, P.V., Bunkoczi, G., Chen, V.B., Davis, I.W., Echols, N., Headd, J.J., Hung, L.W., Kapral, G.J., Grosse-Kunstleve, R.W., *et al.* (2010). PHENIX: a comprehensive Python-based system for macromolecular structure solution. Acta Crystallogr D *66*, 213-221.

Atherton, P., Stutchbury, B., Wang, D.Y., Jethwa, D., Tsang, R., Meiler-Rodriguez, E., Wang, P., Bate, N., Zent, R., Barsukov, I.L., *et al.* (2015). Vinculin controls talin engagement with the actomyosin machinery. Nat Commun *6*, 10038.

Emsley, P., and Cowtan, K. (2004). Coot: model-building tools for molecular graphics. Acta Crystallogr D *60*, 2126-2132.

Evans, P. (2006). Scaling and assessment of data quality. Acta Crystallogr D *62*, 72-82.

Gingras, A.R., Bate, N., Goult, B.T., Patel, B., Kopp, P.M., Emsley, J., Barsukov, I.L., Roberts, G.C., and Critchley, D.R. (2010). Central region of talin has a unique fold that binds vinculin and actin. J Biol Chem *285*, 29577-29587.

Goult, B.T., Gingras, A.R., Bate, N., Roberts, G.C., Critchley, D.R., and Barsukov, I.L. (2008). NMR assignment of the C-terminal actin-binding domain of talin. Biomol NMR Assign *2*, 17-19.

Li, G., Du, X., Vass, W.C., Papageorge, A.G., Lowy, D.R., and Qian, X. (2011). Full activity of the deleted in liver cancer 1 (DLC1) tumor suppressor depends on an LD-like motif that binds talin and focal adhesion kinase (FAK). Proc Natl Acad Sci U S A *108*, 17129-17134.

McCoy, A.J., Grosse-Kunstleve, R.W., Adams, P.D., Winn, M.D., Storoni, L.C., and Read, R.J. (2007). Phaser crystallographic software. J Appl Crystallogr *40*, 658-674.

Painter, J., and Merritt, E.A. (2006). Optimal description of a protein structure in terms of multiple groups undergoing TLS motion. Acta Crystallogr D Biol Crystallogr *62*, 439-450.

Qian, X., Li, G., Vass, W.C., Papageorge, A., Walker, R.C., Asnaghi, L., Steinbach, P.J., Tosato, G., Hunter, K., and Lowy, D.R. (2009). The Tensin-3 protein, including its SH2 domain, is phosphorylated by Src and contributes to tumorigenesis and metastasis. Cancer Cell *16*, 246-258.

Vranken, W.F., Boucher, W., Stevens, T.J., Fogh, R.H., Pajon, A., Llinas, M., Ulrich, E.L., Markley, J.L., Ionides, J., and Laue, E.D. (2005). The CCPN data model for NMR spectroscopy: development of a software pipeline. Proteins *59*, 687-696.

Wider, G., and Dreier, L. (2006). Measuring protein concentrations by NMR spectroscopy. J Am Chem Soc *128*, 2571-2576.
